# Author Correction: Synthesis of a magnetic π-extended carbon nanosolenoid with Riemann surfaces

**DOI:** 10.1038/s41467-022-30135-8

**Published:** 2022-04-22

**Authors:** Jinyi Wang, Yihan Zhu, Guilin Zhuang, Yayu Wu, Shengda Wang, Pingsen Huang, Guan Sheng, Muqing Chen, Shangfeng Yang, Thomas Greber, Pingwu Du

**Affiliations:** 1 Hefei National Laboratory for Physical Sciences at the Microscale, iChEM (Collaborative Innovation Center of Chemistry for Energy Materials), CAS Key Laboratory of Materials for Energy Conversion, Department of Materials Science and Engineering, University of Science and Technology of China, 96 Jinzhai Road, Hefei, Anhui Province 230026 China; 2 Center for Electron Microscopy, State Key Laboratory Breeding Base of Green Chemistry Synthesis Technology and College of Chemical Engineering, Zhejiang University of Technology, Hangzhou, 310014 China; 3 College of Chemical Engineering, Zhejiang University of Technology, 18 Chaowang Road, Hangzhou, Zhejiang Province 310032 China; 4 Physik-Institut, University of Zürich, Winterthurerstrasse 190, CH-8057 Zürich, Switzerland; 5 National Synchrotron Radiation Laboratory, University of Science and Technology of China, 42 Hezuohua South Road, Hefei, Anhui Province 230029 China

**Keywords:** Chemical synthesis, Materials chemistry

Correction to: *Nature Communications* 10.1038/s41467-022-28870-z, published online 9 March 2022.

“The original version of this Article omitted an acknowledgement to the source of Fig. 1c. The following has been added to the end of the caption to Fig. 1: ‘Fig. 1c (left) adapted with permission from ref. 25 (copyright 2016 American Chemical Society), Fig. 1c (middle) adapted with permission from ref. 66 (copyright 2019 American Physical Society), and Fig. 1c (right) adapted with permission from ref. 67 (copyright 2018 American Chemical Society)’. Reference 66 ‘Zhao, Z., Hang. Y., Zhang, Z. & Guo, W. Topological hybrid nodal-loop semimetal in a carbon allotrope constructed by interconnected Riemann surfaces. *Phys. Rev. B* **100**, 115420–115426 (2019)’ and reference 67 ‘Nakakuki, Y., Hirose, T., Sotome, H., Miyasaka, H. & Matsuda, K. Hexa-peri-hexabenzo[7]helicene: homogeneously π-extended helicene as a primary substructure of helically twisted chiral graphenes. *J. Am. Chem. Soc*. **140**, 4317–4326 (2018)’ have been added to the reference list. This has been corrected in the PDF and HTML versions of the Article”.

