# Supplementary material for: Synthesis of a magnetic π-extended carbon nanosolenoid with Riemann surfaces
Source: Nat Commun. 2022 Mar 9;13:1239. doi: 10.1038/s41467-022-28870-z (PMC8907333; doi:10.1038/s41467-022-28870-z)
Supplement: Supplementary file 1 — Supplementary Information [file 41467_2022_28870_MOESM1_ESM.pdf]

Supplementary Information

Synthesis of a magnetic π-extended carbon nanosolenoid with Riemann Surfaces

Jinyi Wang[1,6], Yihan Zhu[2,6], Guilin Zhuang[3,6], Yayu Wu[1], Shengda Wang[1], Pingsen Huang[1], Guan Sheng[2], Muqing Chen[1], Shangfeng Yang[1], Thomas Greber[4], Pingwu Du[1,5]*

[1] Hefei National Laboratory for Physical Sciences at the Microscale, *i*ChEM (Collaborative Innovation Center of Chemistry for Energy Materials), CAS Key Laboratory of Materials for Energy Conversion, Department of Materials Science and Engineering, University of Science and Technology of China, 96 Jinzhai Road, Hefei, Anhui Province, 230026, China

[2] Center for Electron Microscopy, State Key Laboratory Breeding Base of Green Chemistry Synthesis Technology and College of Chemical Engineering, 18 Chaowang Road, Zhejiang University of Technology, Hangzhou, 310014, China

[3] College of Chemical Engineering, Zhejiang University of Technology, 18 Chaowang Road, Hangzhou, Zhejiang Province, 310032, China

[4] Physik-Institut, University of Zürich, Winterthurerstrasse 190, CH-8057 Zürich, Switzerland

[5] National Synchrotron Radiation Laboratory, University of Science and Technology of China, 42 Hezuohua South Road, Hefei, Anhui Province, 230029, China

[6] These authors contributed equally: Jinyi Wang, Yihan Zhu, Guilin Zhuang

*Corresponding author: dupingwu@ustc.edu.cn

Table of Contents

Supplementary Notes.. S3

Supplementary Methods S5

Supplementary References.. S34

Supplementary Notes

All anhydrous solvents and starting chemical reagents for syntheses were purchased from commercial suppliers (Aldrich or Acros) and used without further purification, unless otherwise noted. All moisture- or air-sensitive reactions were carried out in a dry reaction vessel under an inert atmosphere (argon or nitrogen) using standard Schlenk techniques. Preparative column chromatography was performed on silica gel (size 200-300 mesh). Nuclear magnetic resonance (NMR) spectra were recorded at a constant temperature of 25 ℃ using a Bruker BioSpin (^{1}H 400 MHz, ^{13}C 100 MHz) spectrometer. Chemical shifts are expressed in ppm relative to $CHCl_3$ (δ = 7.26 ppm) or tetramethylsilane (TMS, δ = 0.00 ppm) for ^{1}H NMR and $CDCl_3$ (δ = 77.0 ppm) for ^{13}C NMR. Data are reported as follows: chemical shift, multiplicity (s = singlet, d = doublet, t = triplet, m = multiplet, br s = broad signal), coupling constant (Hz), and integration. High-resolution mass spectra were acquired using MALDI-TOF mass techniques and DCTB as the matrix. Gel permeation chromatography (GPC) was carried out using a G1316A PL gel column with a rate of 1.0 min/mL in DMF assured by G1310B Iso.pump and detected by a G1362A differential refractive index detector. Analytical thin-layer chromatographies (TLC) were performed with silica gel HSGF 254. Flash chromatography was performed on silica gel (300~400 mesh). Atomic force microscope (AFM) measurements were performed using a Demension ICON microscope (Bruker) in the tapping mode in a clean room environment. The high-resolution transmission electron microscopy (HRTEM) experiments were carried out on JEM ARM-200F or JEM-2100F microscope operated at 200 kV. The crystal diffraction patterns of the CNS sample were studied by powder X-ray diffraction (XRD, D/max-TTR III) using graphite monochromatized Cu K_α radiation of 1.54178 Å, operating at 40 kV and 200 mA. For the XRD characterization, the CNS was concentrated and dropped on clean glass. Scanning electron microscopy (SEM) was performed on a JSM-6700F instrument. Current density-voltage (*J*-*V*) curves of the

FTO/**P1**/Au and FTO/CNS/Au devices were measured by using a Keithley 2400 source meter with the applied voltage range from -2.0 to 2.0 V in a dark environment. Test the sample thickness on the FTO/**P1**/Au and FTO/CNS/Au devices using a Profiler (KLA-Tencor, USA).

The solid-state NMR experiments were conducted on a Bruker Avance III 400 spectrometer ($\nu_L(^1H)$ = 400 MHz, 9.4 T) using a Bruker 2.5 mm DVT H/F/X probe. The samples were packed in 2.5 mm o.d. ZrO_2 rotos and sealed with Vespel© bottom and top caps. Adamantane was used as an external reference to calibrate the radiofrequency (rf) field strength and chemical shift scale ($\delta(^1H)$ = 1.85 ppm)[1]. The ^1H MAS NMR experiments were recorded at 34 kHz MAS, using a $\pi/2$ pulse length of 2.3 μs (ν_{rf} = 108.6 kHz). The ^1H MAS NMR experiments were recorded with a recycle delay of 5 s. The 2D ^1H-^1H DQ-SQ NMR correlation spectra were recorded with a recycle delay of 1 s. The 2D ^1H-^1H DQ-SQ correlation spectra were recorded employing 4 rotor periods DQ excitation using the Back-to-Back (BaBa) DQ recoupling scheme with a XY16 phase cycle.

Supplementary Methods

Representative Examples of Riemann Surfaces

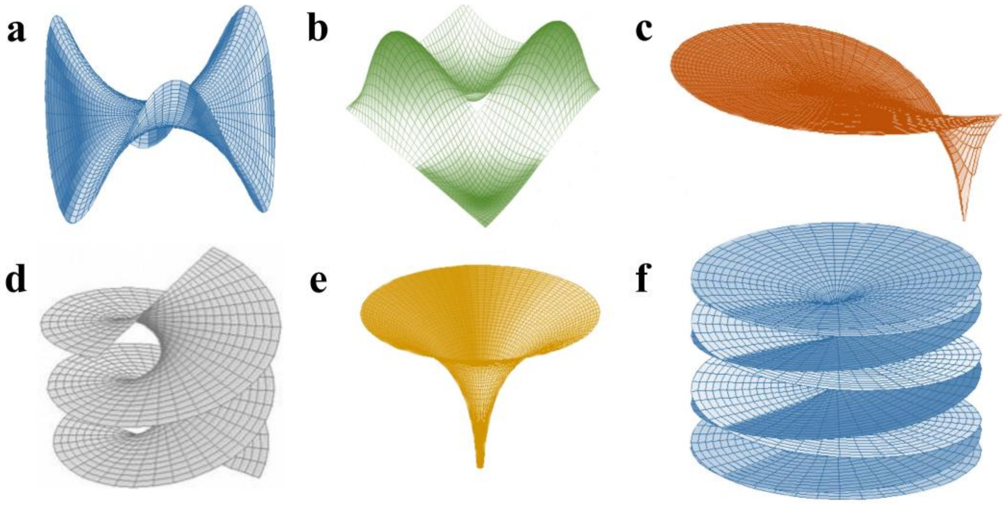

Supplementary Figure 1. a-f Representative examples of Riemann surfaces.

A Conceptual Schematic Diagram of CNS

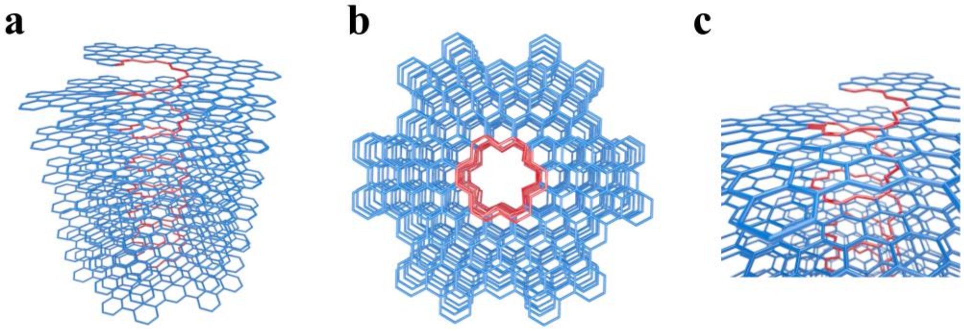

Supplementary Figure 2. A conceptual schematic diagram of CNS views from different angles and areas: **a** tilted side view, **b** top view, **c** side view of molecular chain end enlarged.

Synthetic Procedures

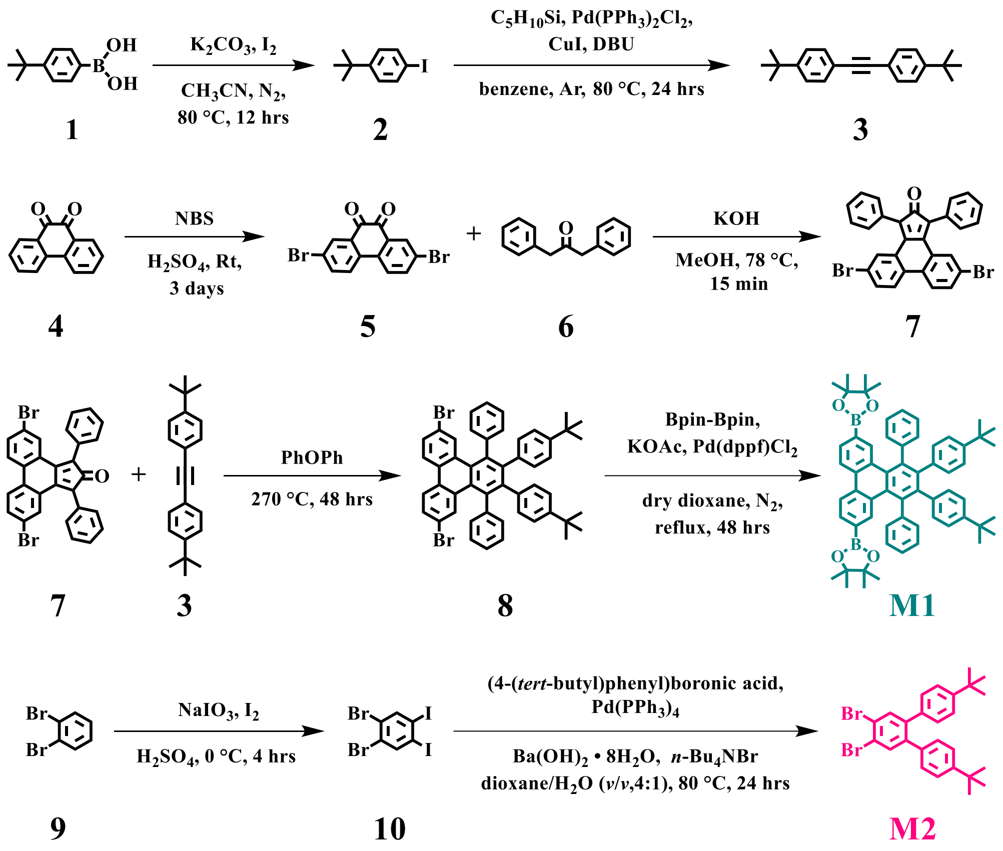

Supplementary Figure 3. Synthesis procedures of compounds **M1** and **M2**.

Synthesis of 1-*tert*-butyl-4-iodobenzene (2), 1,2-bis(4-*tert*-butylphenyl)acetylene (3), 2,7-dibromo-phenanthrene-9,10-dione (5), 5,10-dibromo-1,3-diphenyl-2H-cyclopenta-[*l*]phenanthren-2-one (7), and 1,2-dibromo-4,5-diiodobenzene (10)

The starting materials 1-*tert*-butyl-4-iodobenzene (**2**), 1,2-bis(4-*tert*-butylphenyl)acetylene (**3**), 2,7-dibromo-phenanthrene-9,10-dione (**5**), 5,10-dibromo-1,3-diphenyl-2H-cyclopenta-[*l*]phenanthren-2-one (**7**), and 1,2-dibromo-4,5-diiodobenzene (**10**) were synthesized according to literature reports[2,3,4,5].

4-*tert*-butylphenylboronic acid **1** (8.9 g, 50 mmol), K_2CO_3 (13.8 g, 100 mmol) were dissolved in CH_3CN (200 mL), then I_2 (19.1 g, 75 mmol) was added under

nitrogen atmosphere. Thereafter, the mixture was heated with stirring at 80 °C for 12 hours under nitrogen atmosphere. After cooling down to room temperature, the solvent was removed under reduced pressure, the resulting product was dissolved in PE/CH_2Cl_2 (*v*/*v*, 5:1) and passed through a short silica gel column. After removal of volatiles under reduced pressure to afford **2** as yellow oil (6.0 g, 92%). ^1H NMR ($CDCl_3$, 400 MHz): δ 7.62-7.58 (m, 2H), 7.15-7.11 (m, 2H), 1.29 (s, 9H). The ^1H NMR data are consistent with those reported in the literature[6].

Synthesis of 1,2-bis(4-*tert*-butylphenyl)acetylene (3)

A 100 mL Schlenk flask containing 1-*tert*-butyl-4-iodobenzene **2** (2.60 g, 10 mmol), benzene (50 mL), and a magnetic stirring bar was degassed with argon for 15 min. After that, $Pd(PPh_3)_2Cl_2$ (210 mg, 0.03 mmol), CuI (191 mg, 0.03 mmol) and DBU (9.12 g, 60 mmol) were added under argon, then the mixture was degassed for 5 min. $C_5H_{10}Si$ (490 mg, 5 mmol) and H_2O (72 mg) were transferred to the flask *via* syringe under argon at room temperature. The mixture was then heated to 80 °C in the dark for 24 hours under argon atmosphere. After cooling down to room temperature, water was added and the mixture was extracted with diethyl ether. The combined organic layer was washed with 20% HCl (2 × 20.0 mL), water and brine, and then dried with anhydrous $MgSO_4$, filtered and concentrated under reduced pressure. The residue was purified by silica gel column chromatography to give 1,2-bis(4-*tert*-butylphenyl)acetylene **3** as a white solid (1.3 g, 90%). ^1H NMR ($CDCl_3$, 400 MHz): δ 7.49-7.43 (m, 4H), 7.39-7.33 (m, 4H), 1.33 (s, 18H). The ^1H NMR data are consistent with those reported in the literature[7].

Synthesis of 2,7-dibromo-phenanthrene-9,10-dione (5)

Phenanthrene-9,10-dione **4** (7 g, 33.6 mmol) was dissolved in 98% H_2SO_4 (190 mL) and N-Bromosuccinimide (12.86 g, 72.1 mmol) was added in portions over a period of 40 minutes with continuous stirring. The mixture was vigorously stirred at room temperature for 3 days, then the reaction mixture was poured into a large beaker containing crushed ice (400 g) and left for 1 hour with stirring. The reaction product was filtered and washed thoroughly with H_2O. Finally, the crude product was washed by DMSO to afford 2,7-dibromo-phenanthrene-9,10-dione **5** as orange solid (10.2 g, 83%). ^{1}H NMR (DMSO-d_6, 400 MHz): δ 8.23 (d, J = 8.6 Hz, 2H), 8.05 (d, J = 2.3 Hz, 2H), 7.93 (dd, J_1 = 8.6 Hz, J_2 = 2.3 Hz, 2H). The ^{1}H NMR data are consistent with those reported in the literature[4].

Synthesis of 5,10-dibromo-1,3-diphenyl-2*H*-cyclopenta-[*l*]phenanthren-2-one (7)

2,7-dibromo-phenanthrene-9,10-dione **5** (2.46 g, 6.7 mmol) and 1,3-diphenylpropanone **6** (1.72 g, 8.2 mmol) were added to methanol (15 mL) under stirring. The reaction mixture was heated to 80-85 °C and a solution of potassium hydroxide (305 mg, 5.5 mmol) in methanol (1.5 mL) was added dropwise. The reaction mixture was maintaining at the temperature of 80-85 °C with vigorous stirring for 15 minutes. The cold solution was then filtered and washed with methanol to afford 5,10-dibromo-1,3-diphenyl-2*H*-cyclopenta-[*l*]phenanthren-2-one **7** as a gray-green solid (3.1 g, 85%). ^{1}H NMR ($CDCl_3$, 400 MHz): δ 7.62 (br s, 2H), 7.60 (br s, 2H), 7.50-7.41 (m, 8H), 7.38 (d, 4H). The ^{1}H NMR data are consistent with those reported in the literature[8].

Synthesis of 6,11-dibromo-2,3-bis(4-*tert*-butylphenyl)-1,4-diphenyltriphenylene (8)

To a long neck flask was added 5,10-dibromo-1,3-diphenyl-2*H*-cyclopenta-[*l*]phenanthren-2-one **7** (2 g, 3.7 mmol, 1 equiv.), 1,2-bis(4-*tert*-butylphenyl)acetylene **3** (1.074 g, 3.7 mmol, 1 equiv.) and diphenyl ether (4 mL). The mixture was degassed 5 cycles by pumping and backfilling with nitrogen. The reaction was heated to 270 ℃ for 48 hours and then cooled to 80 ℃ before excessive MeOH was added. The resulting precipitate was collected by filtration and further purified by silica gel column chromatography (eluent: petroleum ether) to afford pure 6,11-dibromo-2,3-bis(4-*tert*-butylphenyl)-1,4-diphenyltriphenylene **8** (2.4 g, 81%). ^{1}H NMR (400 MHz, $CDCl_3$): δ 8.19 (d, J = 8.4 Hz, 2H), 7.66 (d, J = 2.0 Hz, 2H), 7.47 (dd, J_1 = 8.8 Hz, J_2 = 2.0 Hz, 2H), 7.21-7.10 (m, 6H), 7.09-7.00 (m, 4H), 6.86 (d, J = 8.4 Hz, 4H), 6.55 (d, J = 8.4 Hz, 4H), 1.15 (s, 18H). ^{13}C NMR (100 MHz, $CDCl_3$): δ 148.09, 141.95, 141.78, 137.38, 136.78, 132.87, 132.39, 131.86, 130.86, 130.07, 129.44, 129.33, 128.14, 126.60, 124.44, 123.31, 120.02, 34.13, 31.22. HR-MS (MALDI-TOF) m/z calcd. for $C_{50}H_{42}Br_2$ $[M]^+$: 802.1633, found 802.1679.

Synthesis of 6,11-diboryl-2,3-bis(4-*tert*-butylphenyl)-1,4-diphenyltriphenylene (M1)

A 100 mL Schlenk flask containing 6,11-dibromo-2,3-bis(4-*tert*-butylphenyl)-1,4-diphenyltriphenylene **8** (1.0 g, 1.25 mmol, 1 equiv.), bis(pinacolato)diboron (1.27 g, 5.0 mmol, 4 equiv.), dry potassium acetate (736 mg, 7.5 mmol, 6 equiv.), Pd(dppf)Cl_2 (46 mg, 0.063 mmol, 5 mol%), anhydrous 1,4-dioxane (36 mL), and a magnetic stirring bar (oven dried) evacuated and refilled with nitrogen for 5 cycles. The resultant mixture

was heated to 100 ℃ for 48 hours after the flask was sealed. After reaction, the volatiles were removed under reduced pressure and the crude reaction mixture was extracted by CH_2Cl_2. The combined organic layer was washed with H_2O and brine, dried over anhydrous $MgSO_4$, filtered and concentrated under rotary evaporation. The crude product was passed through a short silica gel column with CH_2Cl_2 as the eluent, and recrystallization from CH_2Cl_2/MeOH (*v*/*v*, 1:5) to afford pure 6,11-diboryl-2,3-bis(4-*tert*-butylphenyl)-1,4-diphenyltriphenylene **M1** as white solid (967 mg, 86%). ^{1}H NMR (400 MHz, $CDCl_3$): δ 8.41 (d, J = 8.0 Hz, 2H), 8.14 (d, J = 1.2 Hz, 2H), 7.72 (dd, J_1 = 8.0 Hz, J_2 = 1.2 Hz, 2H), 7.10-7.02 (m, 10H), 6.85 (d, J = 8.4 Hz, 4H), 6.56 (d, J = 8.4 Hz, 4H), 1.21 (s, 24H), 1.16 (s, 18H). ^{13}C NMR (100 MHz, $CDCl_3$): δ 147.68, 142.75, 140.71, 137.90, 137.46, 136.99, 133.38, 132.11, 131.02, 130.51, 127.78, 125.85, 123.12, 122.38, 83.40, 34.10, 31.25, 24.74. HR-MS (MALDI-TOF) m/z calcd. for $C_{62}H_{66}B_2O_4$ $[M]^+$: 896.5147, found 896.5179.

Synthesis of 1,2-dibromo-4,5-diiodobenzene (10)

An oven dried 250 mL round bottom flask containing a fine grounded iodine (3.43 g, 13.5 mmol), $NaIO_3$ (1.34 g, 6.76 mmol), and a magnetic stirring bar. Sulfuric acid (98%, 150 mL) was slowly added to the flask and the mixture was stirred at 40 ℃ for 1 hour in the dark (the flask was shielded by aluminum foil during the entire reaction process). The resulting solution was then cooled to 0 ℃ and 1,2-dibromobenzene **9** (4.00 g, 16.9 mmol) was added in one portion. The mixture was vigorously stirred at 0 °C for 4 hours, and then was poured into a large beaker containing 400 g of ice. The resulting white precipitate was filtered off, washed with much water, dissolved in

$CHCl_3$ and washed with 10% $NaHSO_3$ (aq.), dried over anhydrous MgSO4, and then concentrated by rotary evaporator. The crude product was purified by crystallization from $CHCl_3$/hexanes to afforded pure 1,2-dibromo-4,5-diiodobenzene **10** (6.7 g, 81%) as a white crystalline solid. ^1H NMR (400 MHz, $CDCl_3$): δ 8.05 (s, 2H). The ^1H NMR data are consistent with those reported in the literature[5].

Synthesis of 4',5'-dibromo-4,4''-di-*tert*-butyl-1,1',2',1''-terphenyl (M2)

To a degassed suspension of 1,2-dibromo-4,5-diiodobenzene **10** (2 g, 5.32 mmol, 1 equiv.), (4-*tert*-butylphenyl)boronic acid (1.89 g, 10.64 mmol, 2 equiv.), $Ba(OH)_2.8H_2O$ (6.72 g, 21.29 mmol, 4 equiv.), *n*-Bu_4NBr (172 mg, 0.53 mmol, 10 mol%), and $Pd(PPh_3)_4$ (184.42 mg, 0.16 mmol, 3 mol%) were added in dioxane/H_2O (52 mL : 13 mL). The mixture was bubbled with argon for 15 minutes, then the flask was sealed and heated to 80 ℃ for 24 hours. After reaction, 1 mol/L HCl (40 mL) was added and most of dioxane was removed by rotary evaporation, the product was extracted with CH_2Cl_2 and washed twice with brine, dried over anhydrous $MgSO_4$ and concentrated under rotary evaporation. The crude product was passed through a silica gel column chromatography with petroleum ether as the eluent, and recrystallization from CH_2Cl_2/MeOH (*v*/*v*, 1:10) to afford pure 4',5'-dibromo-4,4''-di-*tert*-butyl-1,1',2',1''-terphenyl **M2** as white solid (1.8 g, 54%). ^1H NMR (400 MHz, $CDCl_3$): δ 7.66 (s, 2H), 7.22 (d, J = 8.4 Hz, 4H), 7.01 (d, J = 8.8 Hz, 4H), 1.28 (s, 18H). ^{13}C NMR (100 MHz, $CDCl_3$) δ 150.16, 141.12, 136.23, 135.19, 129.13, 124.92, 123.13, 34.46, 31.26. HR-MS (MALDI-TOF) m/z calcd. for $C_{26}H_{28}Br_2$ $[M]^+$: 500.0537, found 500.1273.

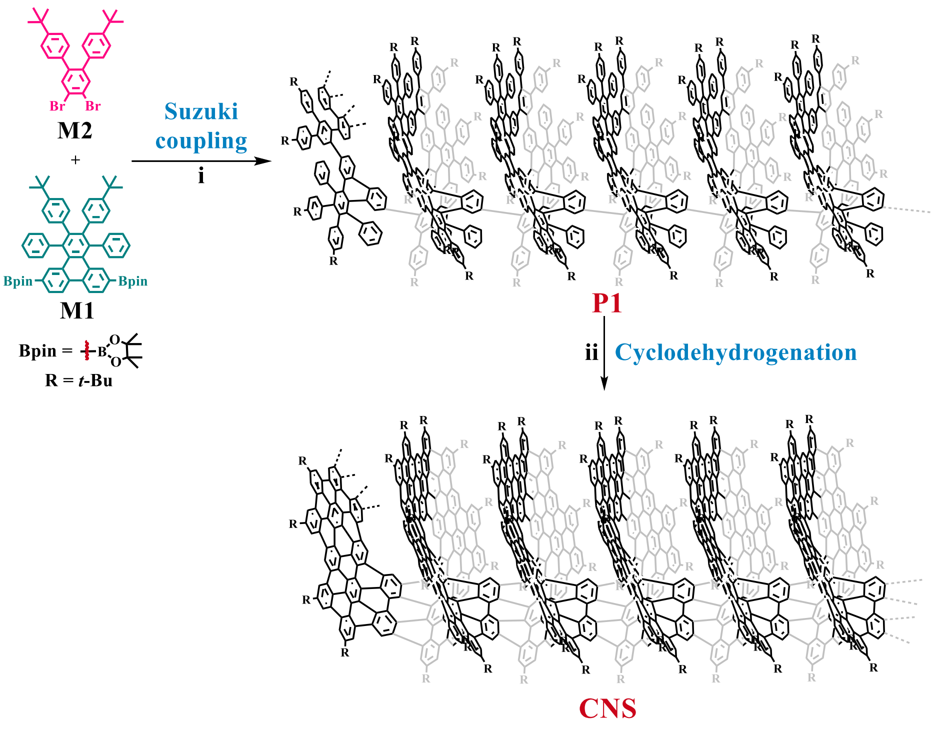

Supplementary Figure 4. Synthesis procedures of compounds **P1** and CNS. Reagents and conditions: (**i**) **M1** (1.0 equiv.), **M2** (1.0 equiv.), K_2CO_3 (10 equiv.), Aliquat 336 (5 mol%), $Pd(PPh_3)_4$ (10 mol%), Ar, toluene/H_2O (*v*/*v*, 5:1), 110 ℃, 5 days; (**ii**) **P1** (1.0 equiv.), DDQ (14 equiv.), TfOH, Ar, anhydrous CH_2Cl_2, 0 ℃, 15 hours.

Synthesis of compound P1

To a degassed suspension of **M1** (403.57 mg, 0.45 mmol, 1 equiv.), **M2** (225.14 mg, 0.45 mmol, 1 equiv.), K_2CO_3 (621.92 mg, 4.5 mmol, 10 equiv.), Aliquat 336 (9.1 mg, 0.023 mmol, 5 mol%) and $Pd(PPh_3)_4$ (52 mg, 0.045 mmol, 10 mol%) were added in toluene/H_2O (20 mL : 4 mL). The reaction mixture was heated to 110 ℃ for 5 days after being degassed by argon bubbling for 15 minutes. After the mixture was poured into MeOH, the resulting gray residue was collected by filtration, and then the residue was dissolved in CH_2Cl_2 and passed through a short silica gel column. Then CH_2Cl_2 was removed under rotary evaporation to afford a crude product, further purification by

recrystallization from CH_2Cl_2/MeOH (*v*/*v*, 1:2) gave the target polymer **P1** as a pale white solid (395 mg, 89%).

Synthesis of compound CNS

A solution of **P1** (380 mg, 0.386 mmol) and DDQ (1.23g, 5.40 mmol) in 40 mL of anhydrous CH_2Cl_2 was degassed by argon bubbling for 20 minutes. The mixture was cooled with an ice bath and then was added TfOH (1.9 mL). After stirring at 0 ℃ for another 15 hours, the reaction was quenched with saturated $NaHCO_3$ solution. The organic phase was separated, washed with saturated $NaHCO_3$ solution and brine, dried over anhydrous magnesium sulfate and evaporated. The crude product was dissolved in THF and reprecipitated with MeOH. This resulting precipitate was then collected by centrifugation (10.0 Krpm, 2 minutes) and washed by a mixture of THF and MeOH (1:1 by volume) and HCl (1.0 M). This process was repeated four times to obtain the target compound CNS as a dark black solid (287 mg, 77%).

GPC Curve of P1

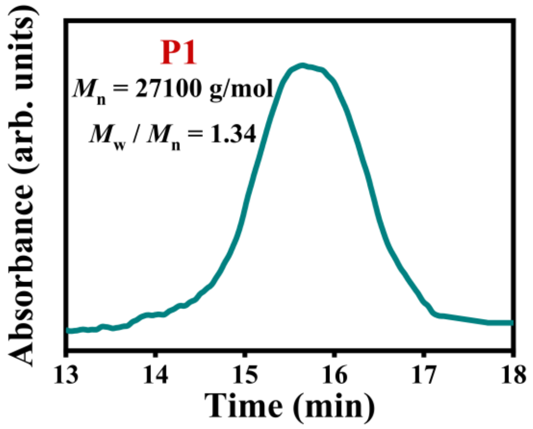

Supplementary Figure 5. GPC curve of **P1** using DMF as the solvent.

Solid-State NMR Characterization of P1 and CNS

Supplementary Figure 6 shows the ^{1}H MAS NMR and 2D ^{1}H-^{1}H DQ-SQ NMR spectra for **P1** and CNS. The comparison of the ^{1}H MAS NMR spectra (Supplementary Figure 6a) of **P1** and CNS demonstrates an obvious increase in ^{1}H line width after graphitization. Supplementary Figures 6b-6c show the solid-state 2D ^{1}H-^{1}H DQ-SQ NMR correlation spectra of **P1** and CNS. The spectrum of **P1** in Supplementary Figure 6b has the expected ^{1}H-^{1}H auto-correlation signals (SQ = 0.7/DQ = 1.4 ppm and SQ = 6.6/DQ = 13.2 ppm). The ^{1}H-^{1}H cross-correlation signals can be generated intramolecularly since the *tert*-butyl groups are connected to a phenyl group. For CNS, the ^{1}H-^{1}H auto-correlation signals of the aliphatic protons can be straightforwardly assigned (SQ = 2.0/DQ = 4.0 ppm). There are almost no ^{1}H-^{1}H auto-correlation signals of the aromatic protons, which is consistent with the feature that the aromatic protons in CNS are far away from each other. The ^{1}H-^{1}H cross-correlation between the *tert*-butyl groups and the aromatic protons (SQ = 9.7/DQ = 11.7 ppm) can also result from intramolecular interactions.

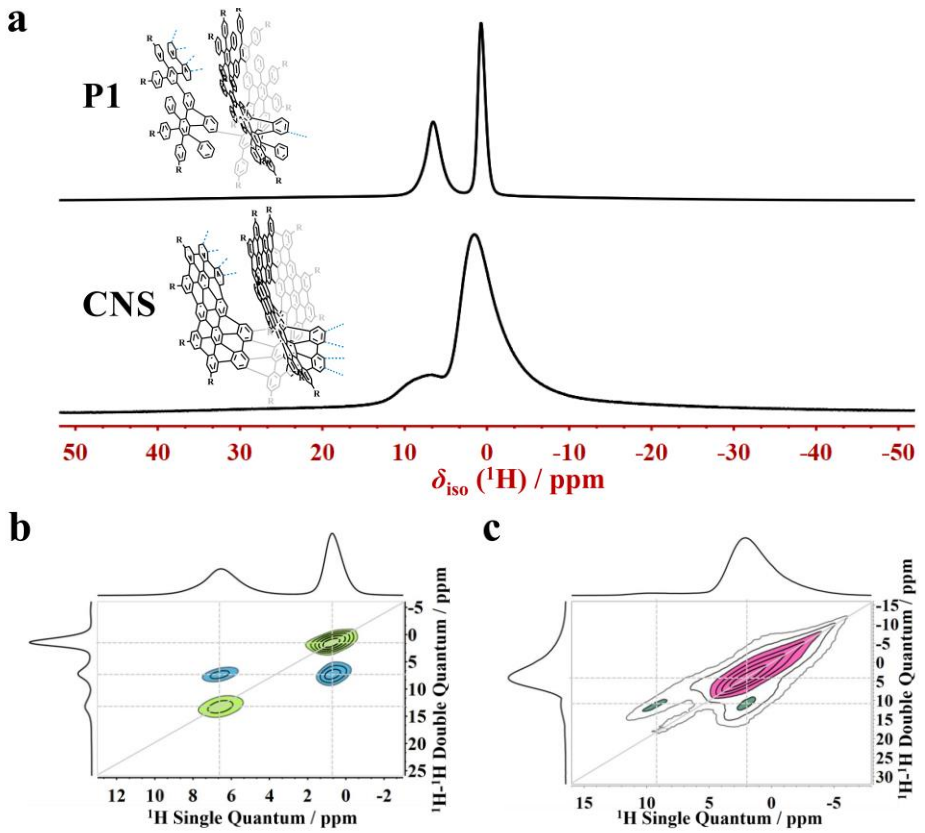

Supplementary Figure 6. a ^{1}H MAS NMR spectra of **P1** (top) and CNS (bottom). The 2D ^{1}H-^{1}H DQ-SQ correlation spectra shown for **P1** (**b**) and CNS (**c**) were recorded employing 4 rotor periods DQ excitation using the Back-to-Back (BaBa) DQ recoupling scheme with an XY16 phase cycle.

XRD Measurement of CNS

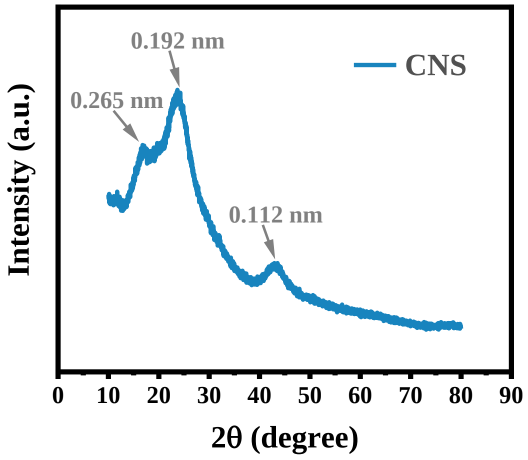

Supplementary Figure 7. XRD patterns of CNS.

HRTEM Measurements of CNS

The stock solution of CNS in dry THF was prepared, which was then diluted with anhydrous ethanol. Samples for the HRTEM measurements of CNS were prepared by dropping stock solution of CNS on a copper grid at room temperature (ca. 25 °C), and the solvents were slowly evaporated under air atmosphere. The experiments were operated at 200 kV.

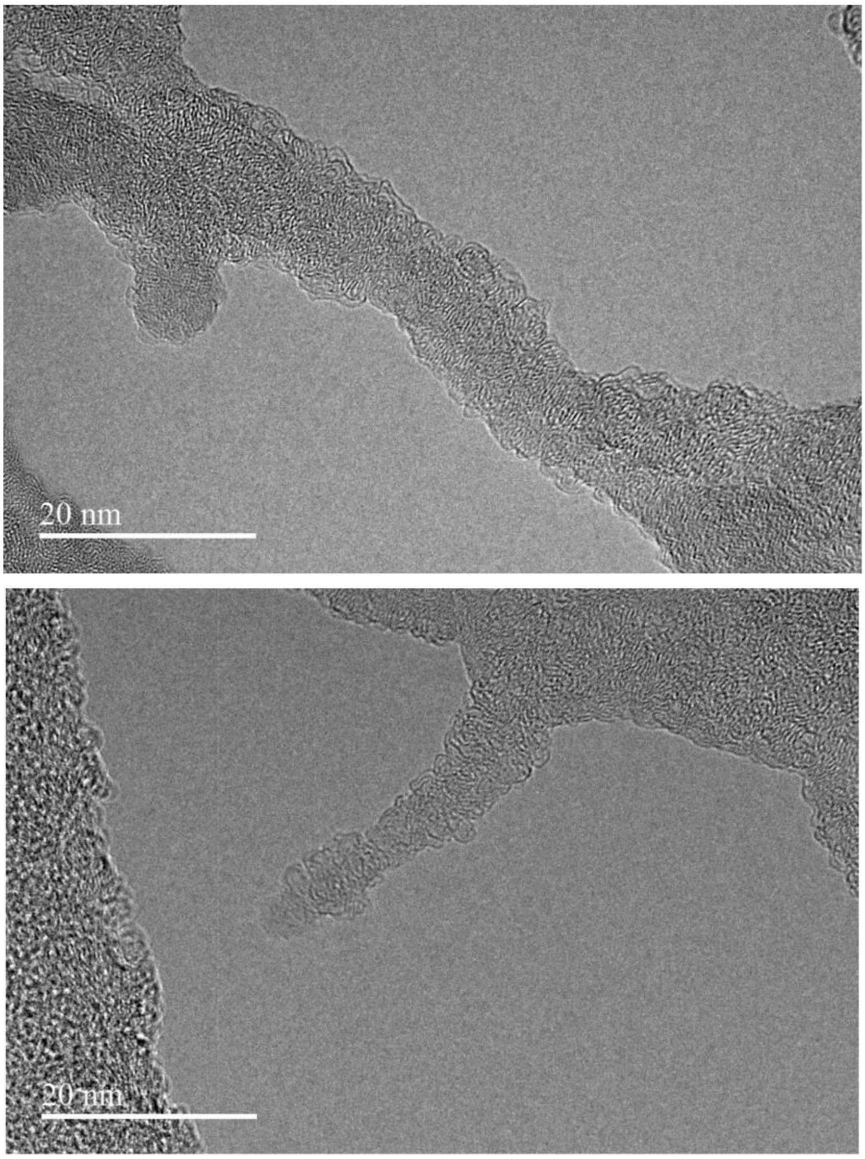

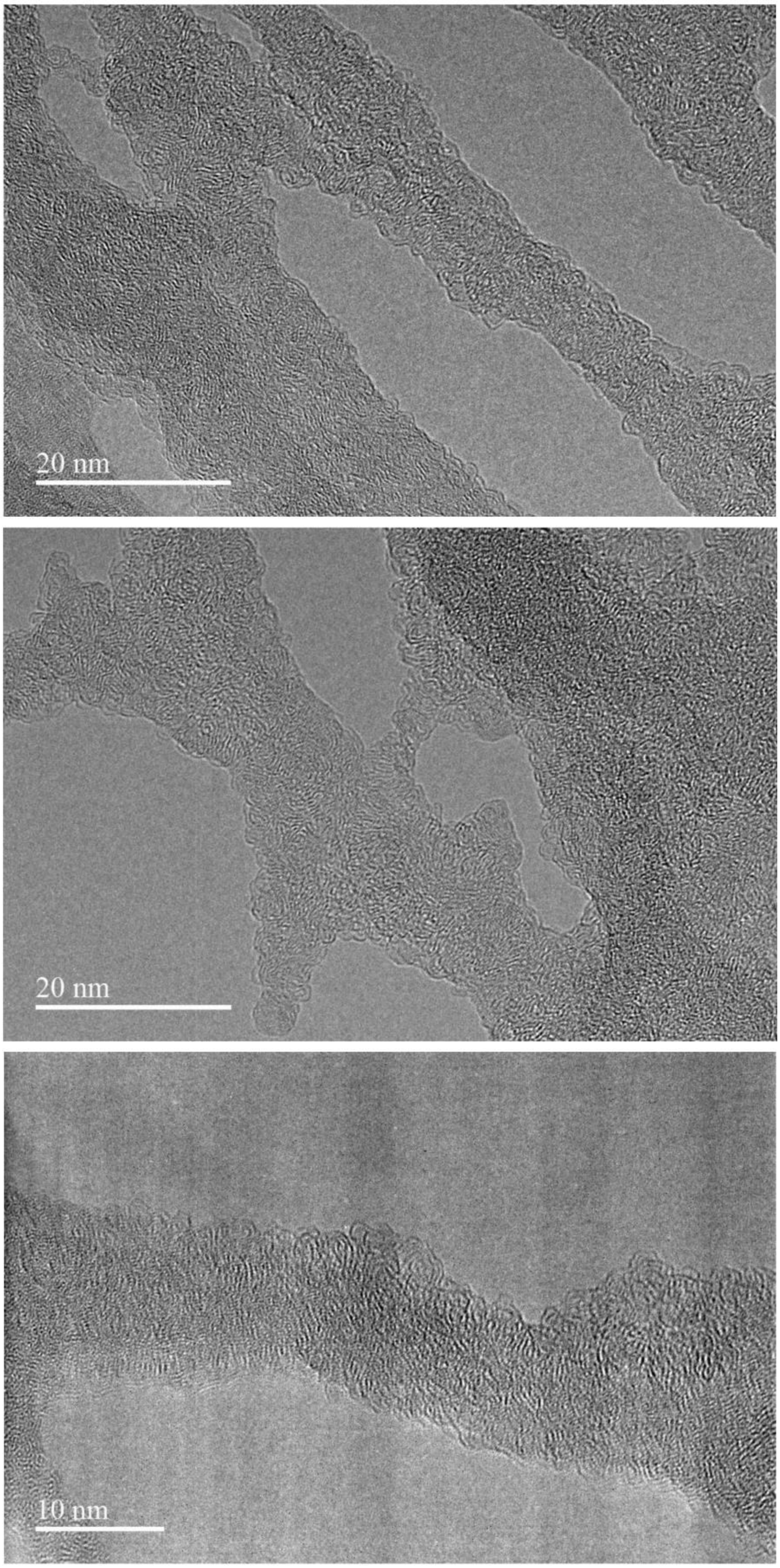
20 nm
20 nm
10 nm

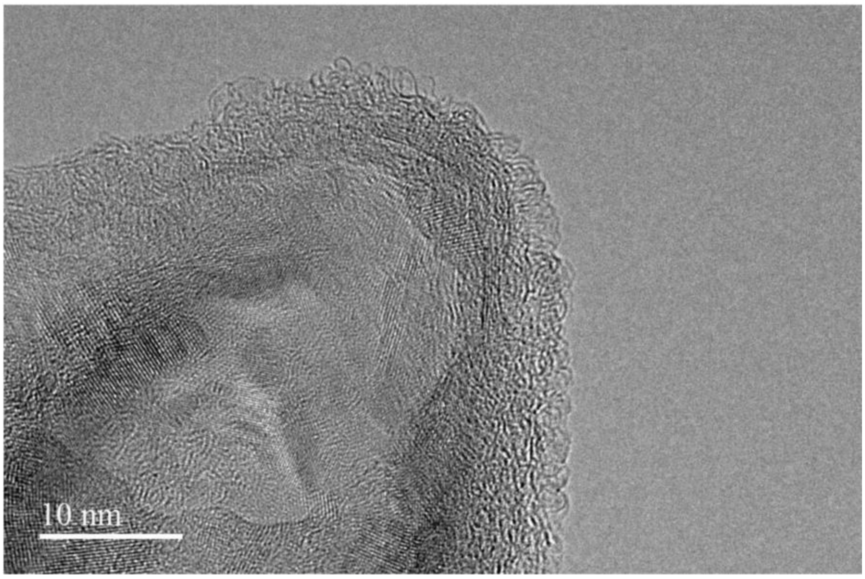

Supplementary Figure 8. Spherical aberration-corrected HRTEM images with different fields of view and different resolutions of CNS. The experiment was carried out on JEM ARM-200F microscope operated at 200 kV.

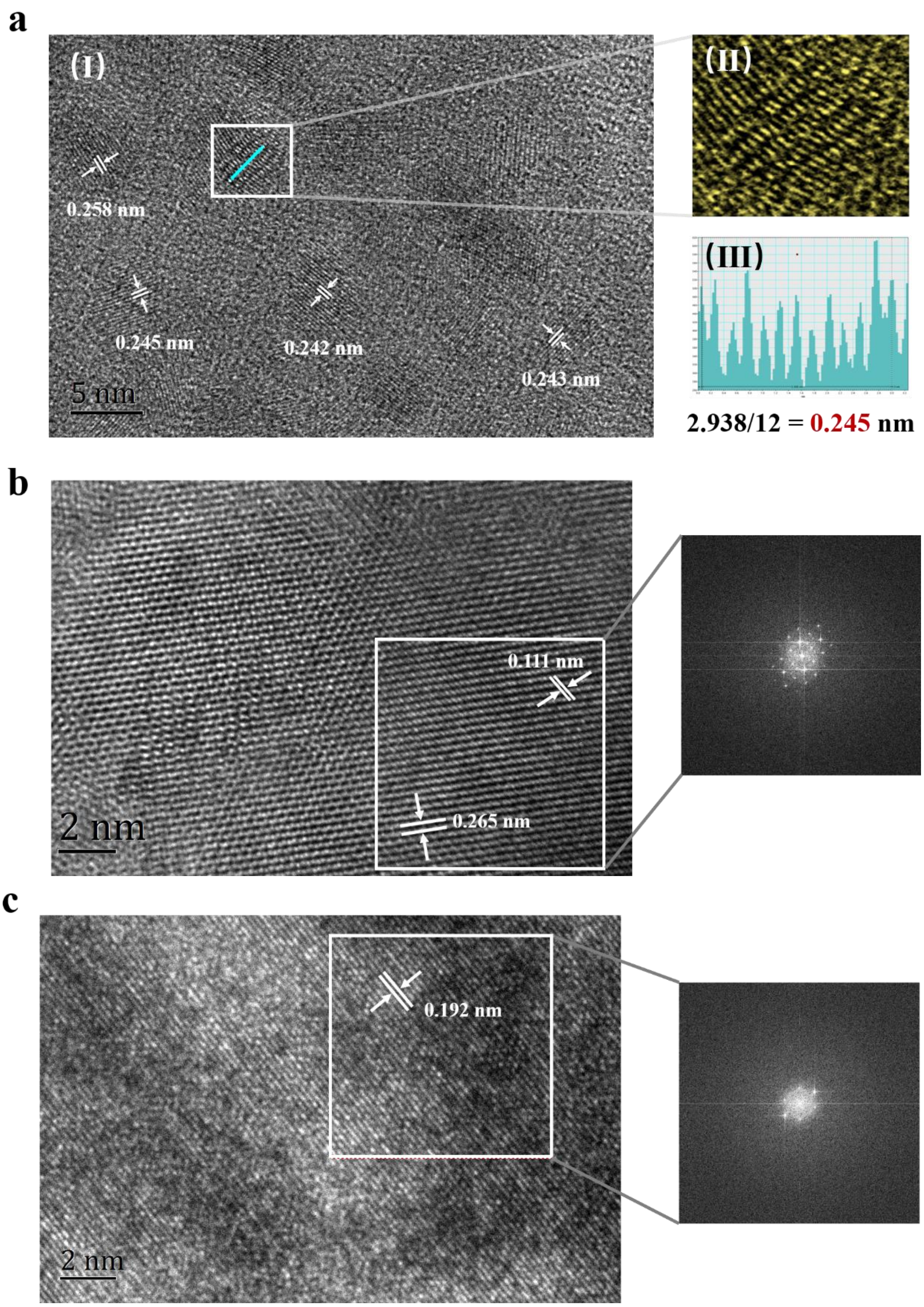

Supplementary Figure 9. **a-c** HRTEM images with different fields of view and different resolutions of CNS. The experiment was carried out on JEM-2100F microscope operated at 200 kV.

Low-Dose iDPC-STEM Measurements of CNS

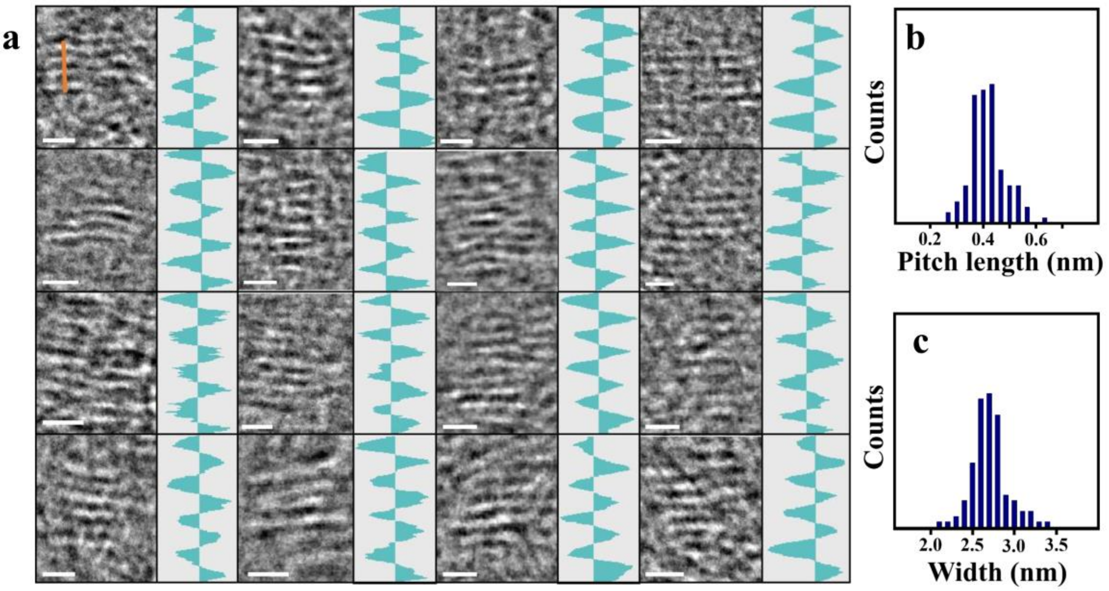

Supplementary Figure 10. **a** Low-dose iDPC-STEM images with different fields of view of CNS samples from different batches. Scale bars: 1 nm. Statistical analysis of helical pitch length (**b**) and width (**c**) of CNS samples shows that measured helical pitch length and width values are mainly distributed at 0.40 ± 0.03 nm and 2.7 ± 0.2 nm, respectively.

SEM Measurements of CNS

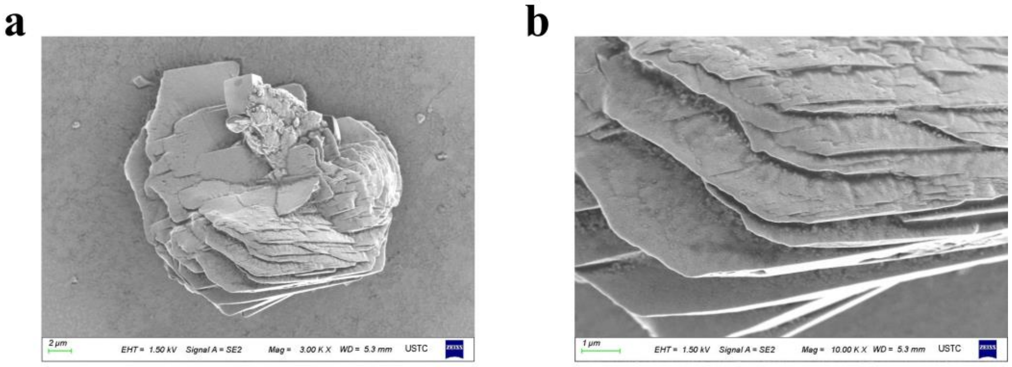

Supplementary Figure 11. a-b SEM images of self-assembled CNS at different resolutions and views.

EPR Measurements of P1 and CNS

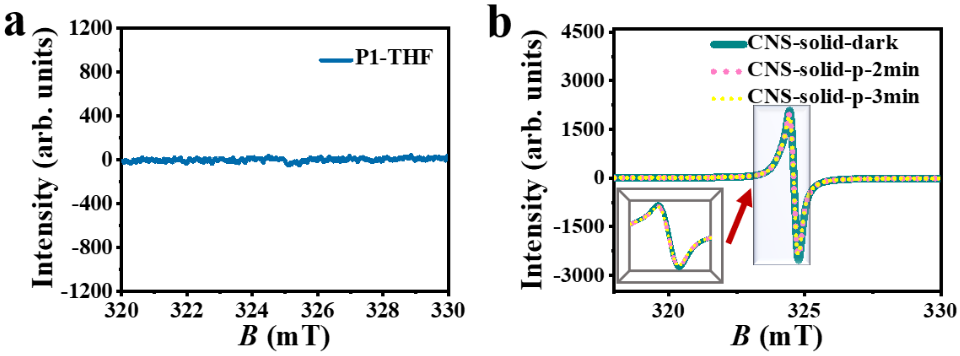

Supplementary Figure 12. a EPR spectrum of THF solution of **P1** recorded at room temperature. **b** EPR spectra of solid powder of CNS under illumination for 2 min and 3 min under 500 W xenon lamp.

J-V Profiles of a Cast Film of P1 and CNS

The device structure is FTO/**P1**/Au or FTO/CNS/Au. **P1** or CNS film is deposited on FTO (Fluorine-Doped Tin Oxide) substrate by spin-coating the THF solution of P1 or CNS. Then, 70 nm Au electrode was deposited on it by vacuum evaporation. The effective area measured is 0.12 cm^2.

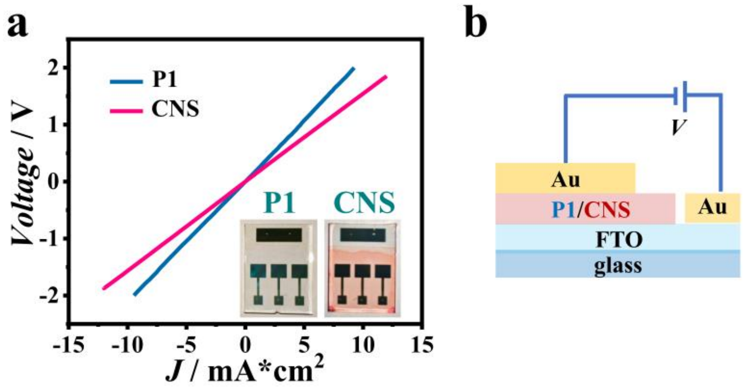

Supplementary Figure 13. a *J-V* profiles of a cast film of **P1** and CNS. Inset: The FTO/**P1**/Au and FTO/CNS/Au devices for an electrical conductivity test. The samples thickness is ~161 nm for **P1** and ~265 nm for the CNS when the average value is taken after three tests. **b** Schematic diagram of the FTO/**P1**/Au or FTO/CNS/Au device.

Physical Characterizations of Intermediate Compounds

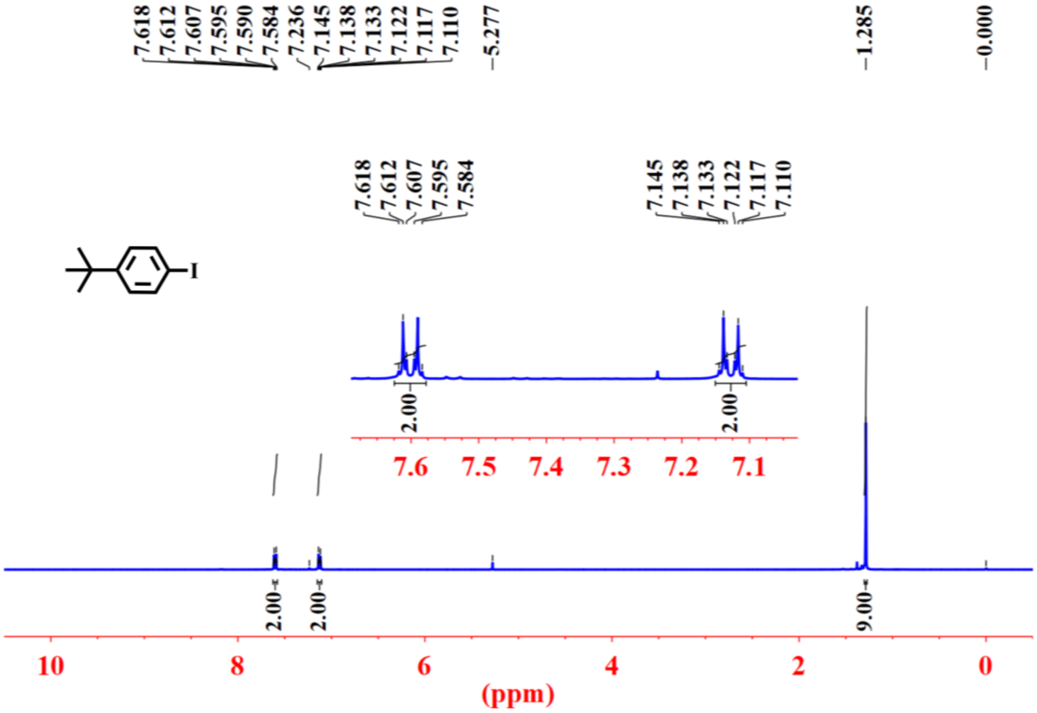

Supplementary Figure 14. ^{1}H NMR spectrum of 1-*tert*-butyl-4-iodobenzene (**2**) in $CDCl_3$.

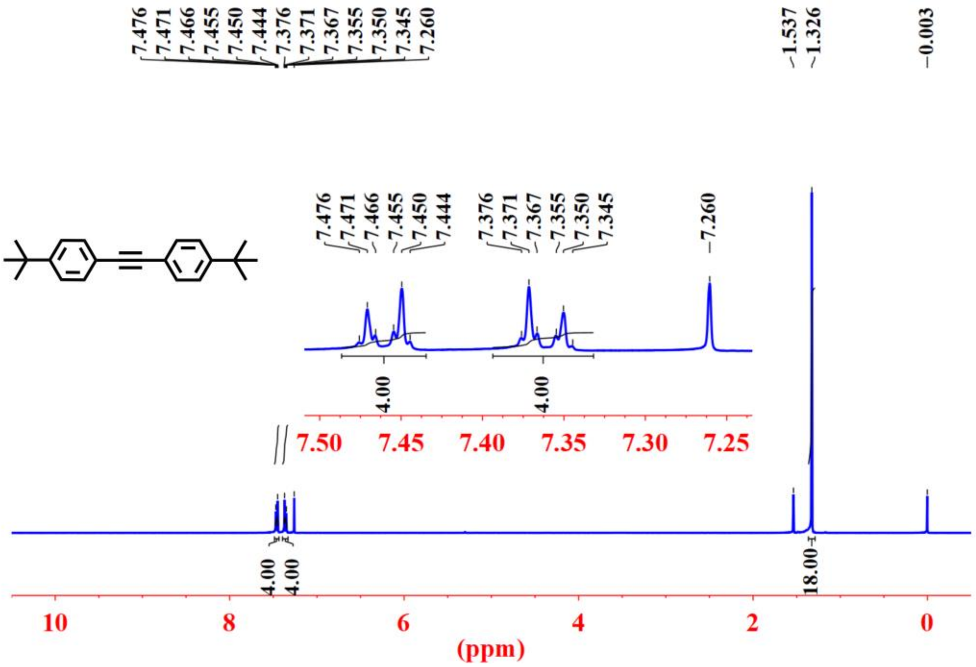

Supplementary Figure 15. ^{1}H NMR spectrum of 1,2-bis(4-*tert*-butylphenyl)acetylene (**3**) in $CDCl_3$.

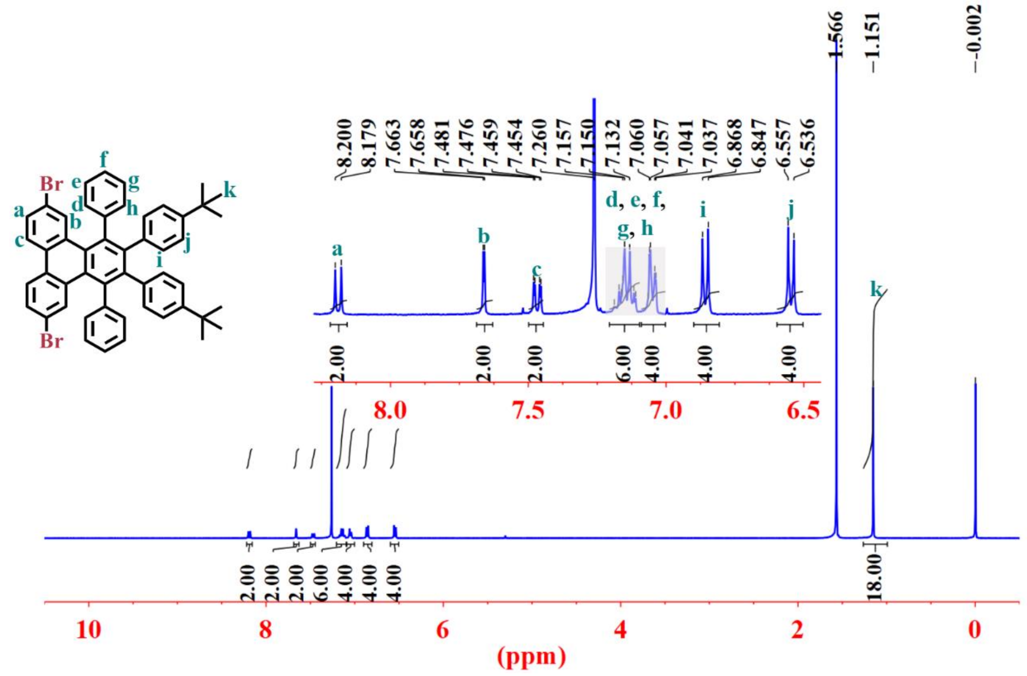

Supplementary Figure 16. ^{1}H NMR spectrum of 6,11-dibromo-2,3-bis(4-*tert*-butylphenyl)-1,4-diphenyltriphenylene (**8**) in $CDCl_3$.

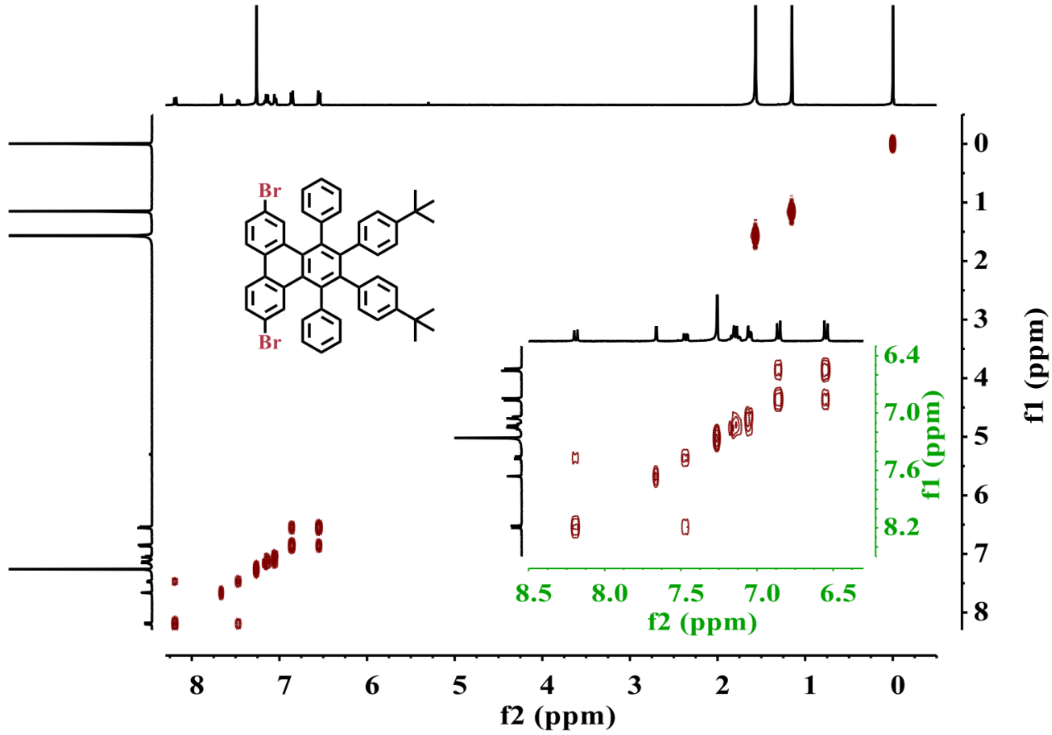

Supplementary Figure 17. 2D ^{1}H-^{1}H COSY NMR spectrum of 6,11-dibromo-2,3-bis(4-*tert*-butylphenyl)-1,4-diphenyltriphenylene (**8**) in $CDCl_3$.

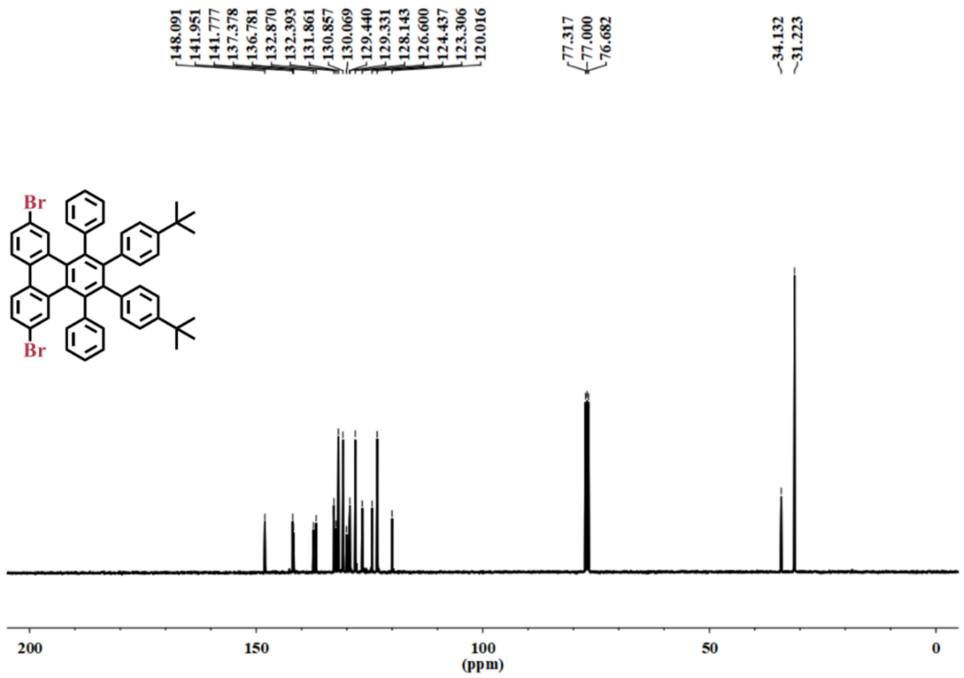

Supplementary Figure 18. ^{13}C NMR spectrum of 6,11-dibromo-2,3-bis(4-*tert*-butylphenyl)-1,4-diphenyltriphenylene (**8**) in $CDCl_3$.

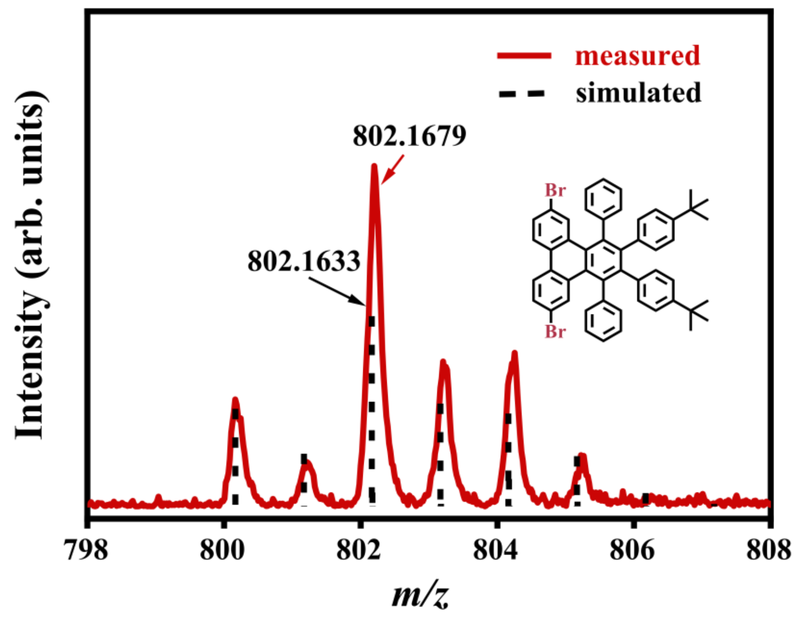

Supplementary Figure 19. HR-MS (MALDI-TOF) data for 6,11-dibromo-2,3-bis(4-*tert*-butylphenyl)-1,4-diphenyltriphenylene (**8**).

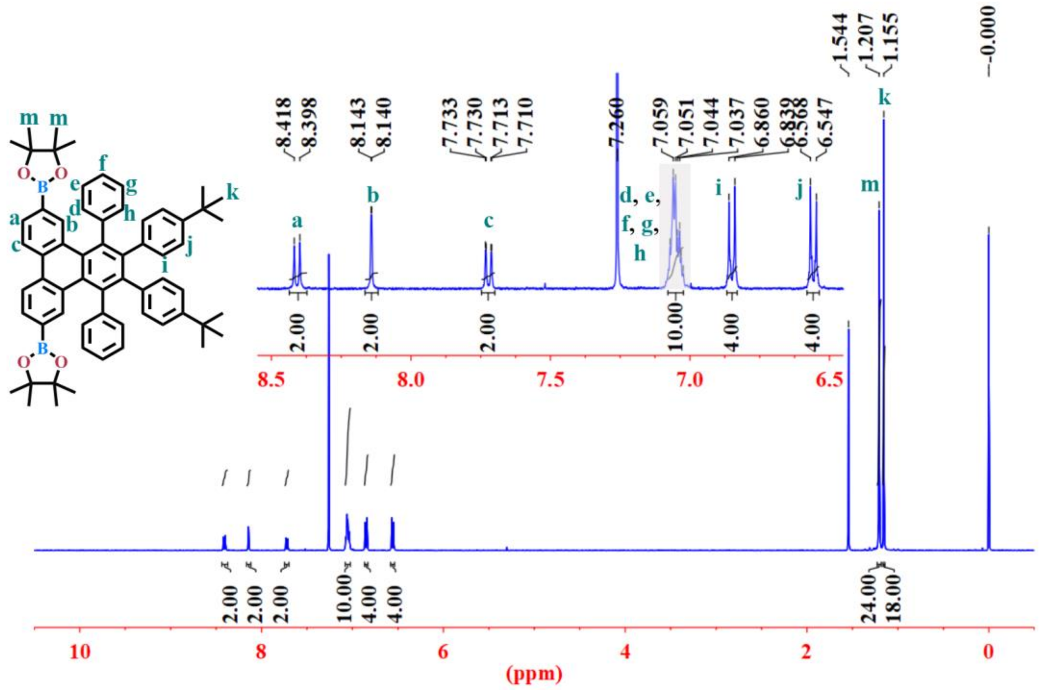

Supplementary Figure 20. ^{1}H NMR spectrum of 6,11-diboryl-2,3-bis(4-*tert*-butylphenyl)-1,4-diphenyltriphenylene (**M1**) in $CDCl_3$.

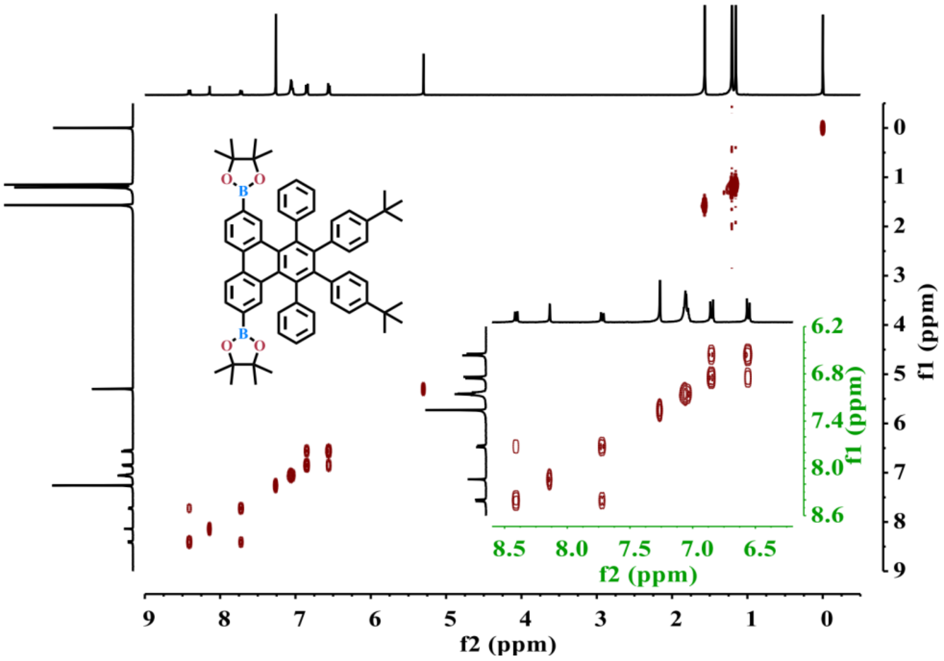

Supplementary Figure 21. 2D ^{1}H-^{1}H COSY NMR spectrum of 6,11-diboryl-2,3-bis(4-*tert*-butylphenyl)-1,4-diphenyltriphenylene (**M1**) in $CDCl_3$.

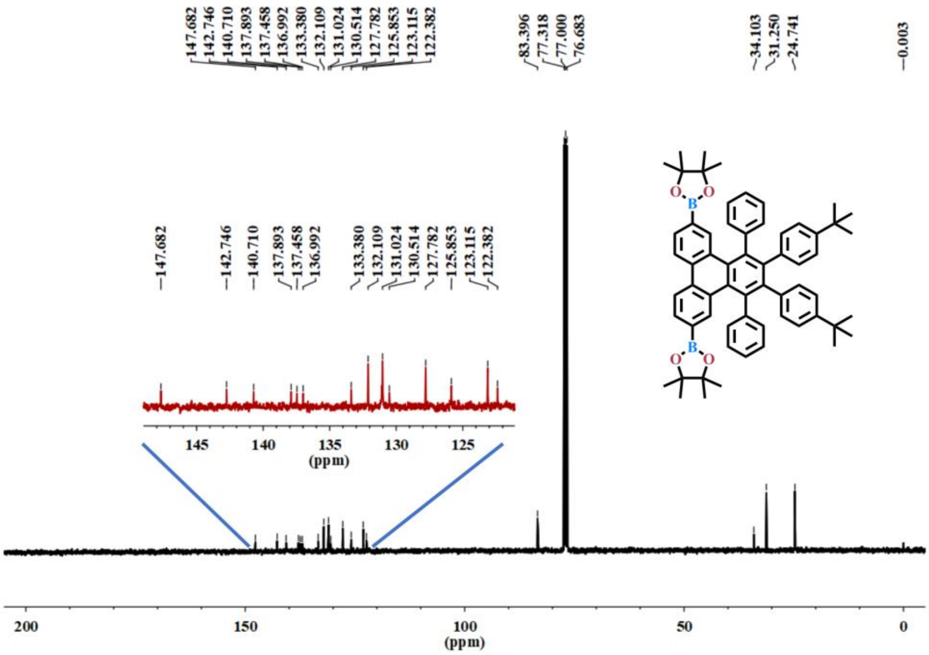

Supplementary Figure 22. ^{13}C NMR spectrum of 6,11-diboryl-2,3-bis(4-*tert*-butylphenyl)-1,4-diphenyltriphenylene (**M1**) in $CDCl_3$.

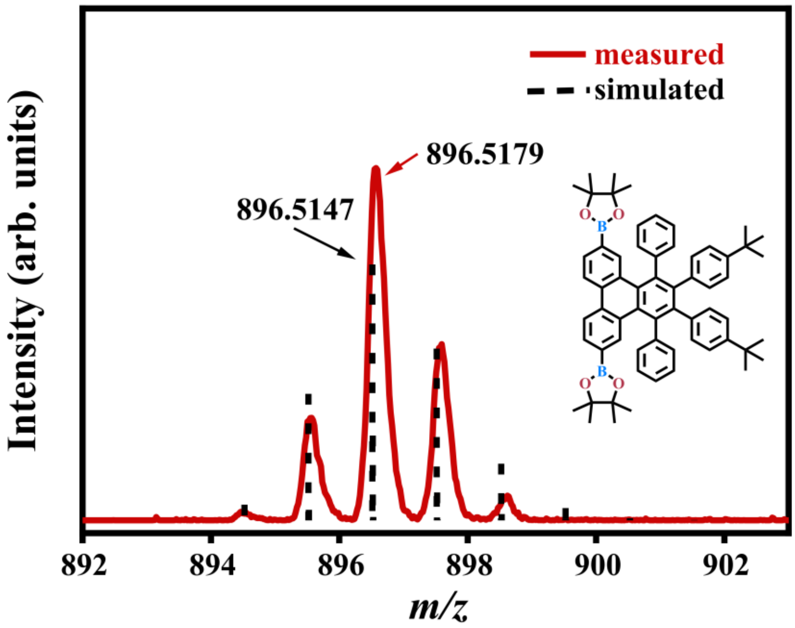

Supplementary Figure 23. HR-MS (MALDI-TOF) data for 6,11-diboryl-2,3-bis(4-*tert*-butylphenyl)-1,4-diphenyltriphenylene (**M1**).

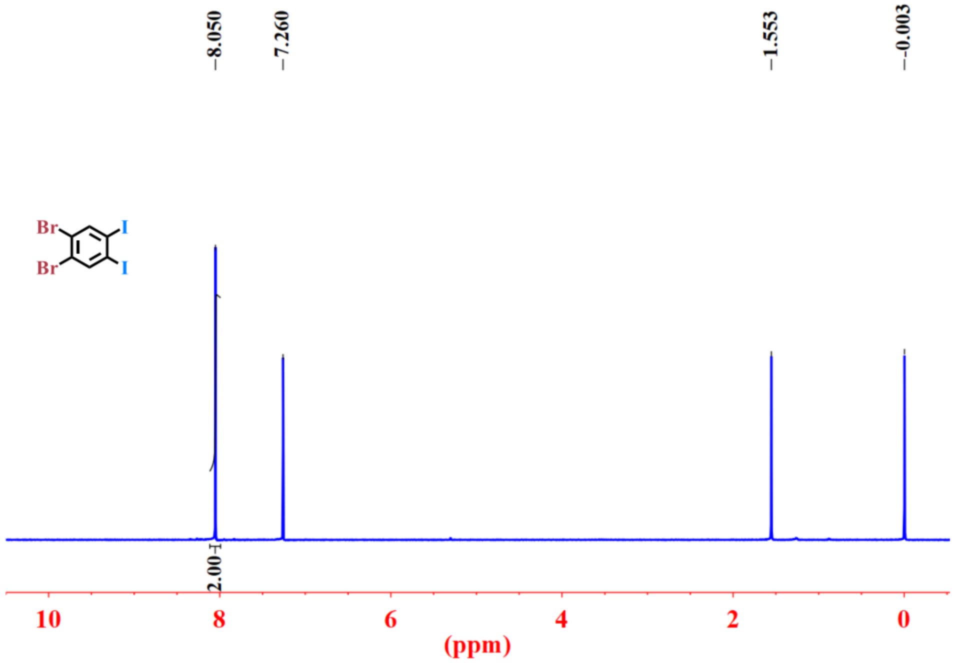

Supplementary Figure 24. ^{1}H NMR spectrum of 1,2-dibromo-4,5-diiodobenzenein (**10**) in $CDCl_3$.

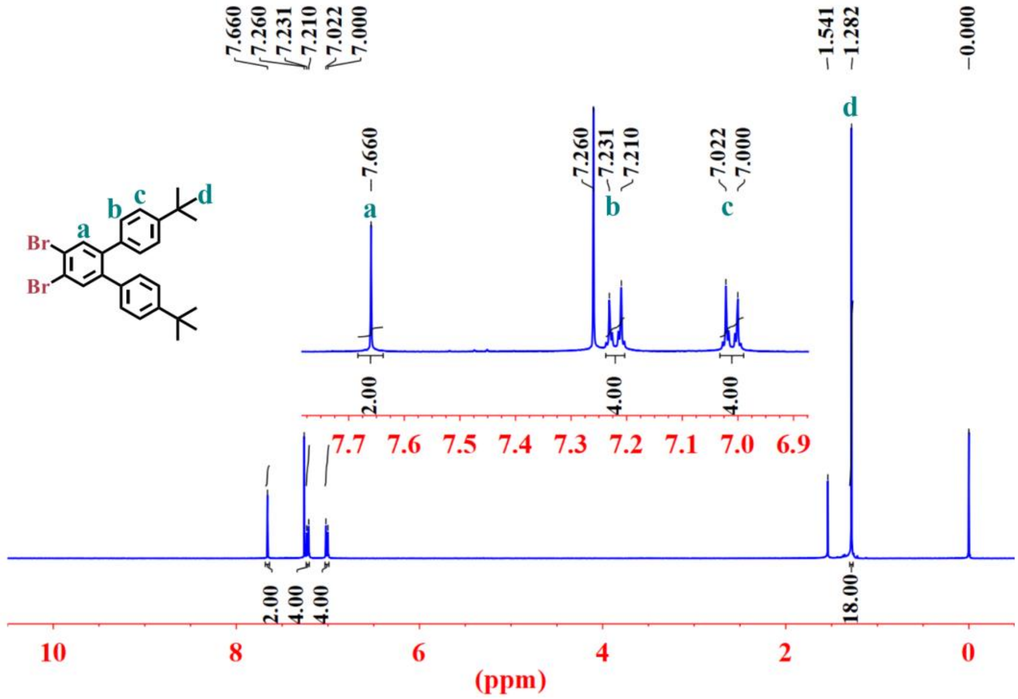

Supplementary Figure 25. ^{1}H NMR spectrum of 4',5'-dibromo-4,4''-di-*tert*-butyl-1,1':2',1''-terphenyl (**M2**) in $CDCl_3$.

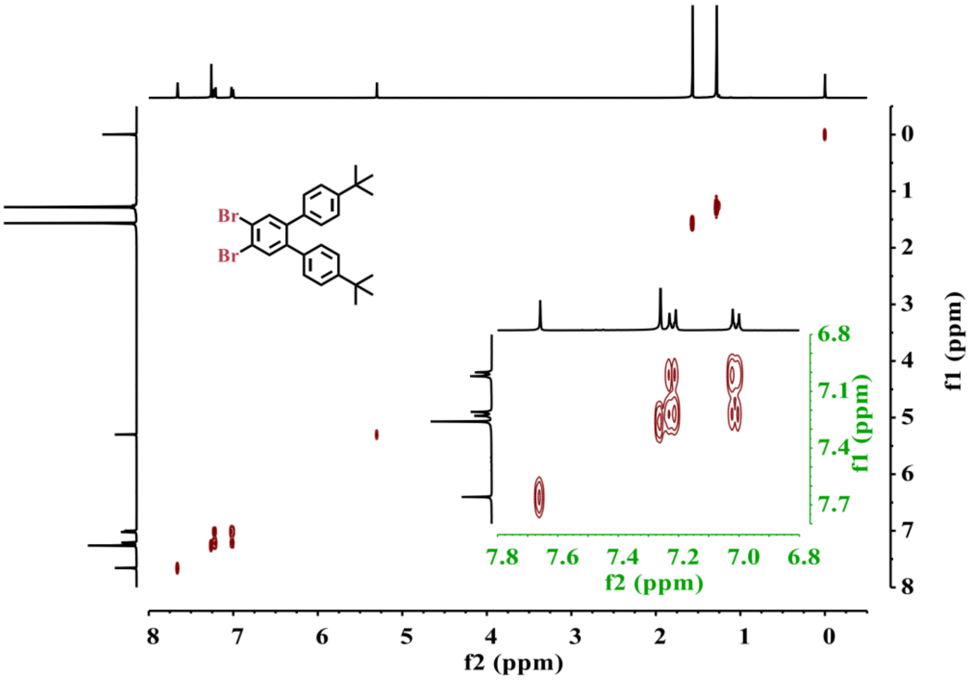

Supplementary Figure 26. 2D ^{1}H-^{1}H COSY NMR spectrum of 4',5'-dibromo-4,4''-di-*tert*-butyl-1,1':2',1''-terphenyl (**M2**) in $CDCl_3$.

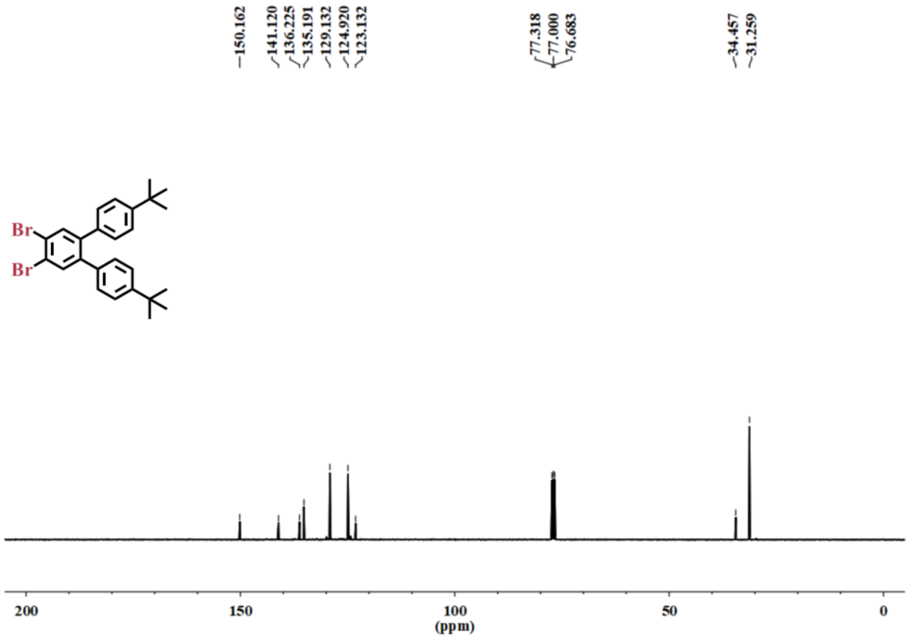

Supplementary Figure 27. ^{13}C NMR spectrum of 4',5'-dibromo-4,4''-di-*tert*-butyl-1,1':2',1''-terphenyl (**M2**) in $CDCl_3$.

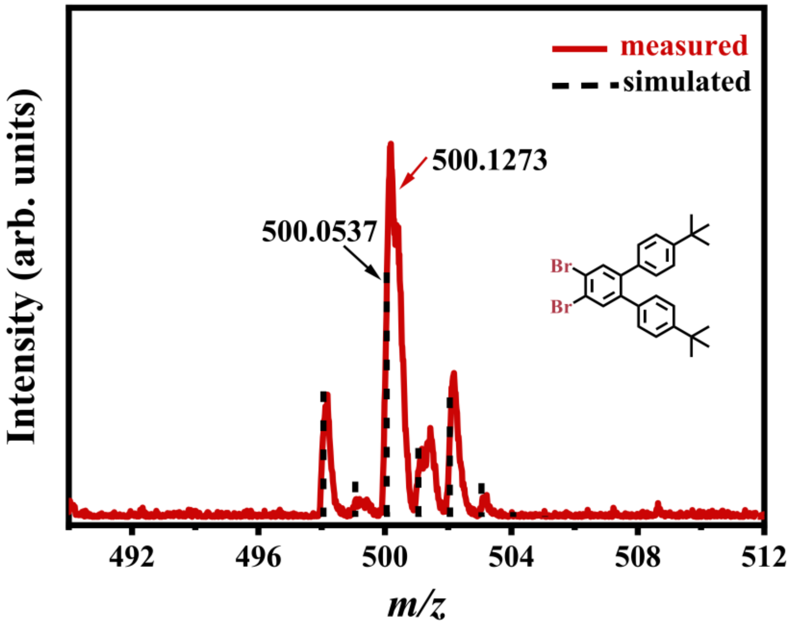

Supplementary Figure 28. HR-MS (MALDI-TOF) data for 4',5'-dibromo-4,4''-di-*tert*-butyl-1,1':2',1''-terphenyl (**M2**).

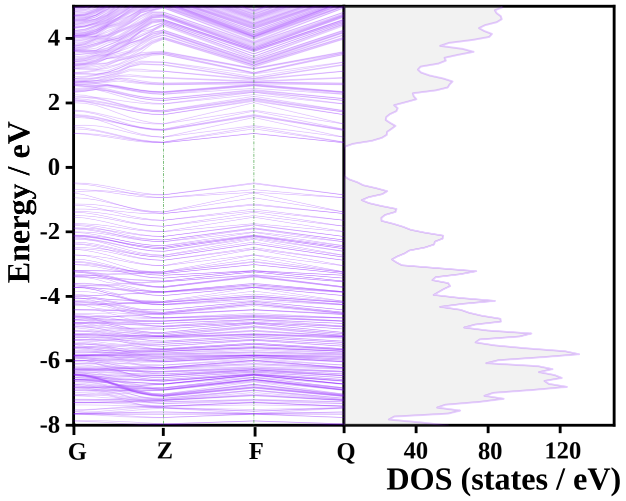

Supplementary Figure 29. Band structure with high-symmetry points (G: 0.0, 0.0, 0.0; Z: 0.0, 0.0, 0.5; F: 0.0, 0.5, 0.0; Q: 0.0, 0.5, 0.5) and density of states of CNS.

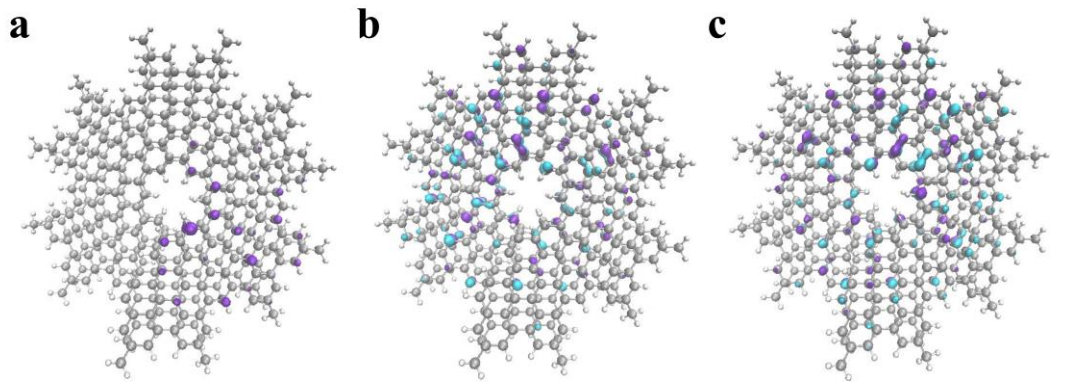

Supplementary Figure 30. a Spin density of CNS. **b-c** Single-occupied molecular orbitals of CNS.

Supplementary Table 1. Coordinate and lattice parameters of CNS polymers.

Coordinate of CNS unit cell with lattice parameters (a = 41.6219 Å, b = 41.3540 Å, c = 4.11810 Å, α = 90.1662°, β = 88.0819°, γ = 90.3638°)							
H	22.709957	21.654968	3.515878	C	26.725300	30.591186	0.359150
H	28.580250	28.706365	4.075589	C	26.250281	31.893766	0.512212
H	33.525380	19.779001	2.806301	C	24.882769	32.122670	0.363875
H	34.261114	23.975367	2.881498	C	23.980207	31.087435	0.079561
H	32.353791	18.361535	3.241031	C	18.726136	20.806790	2.269441
H	33.035835	16.619907	3.304622	C	18.279061	22.123828	2.429808
H	31.016106	11.013216	3.183876	C	16.882805	22.386488	2.310911
H	22.666309	33.467750	0.314876	C	15.976544	21.298406	2.152372
H	18.873701	32.019012	3.648839	C	19.197660	23.227415	2.701362
H	27.792237	30.416444	0.501028	C	18.703698	24.564336	2.724104
H	24.520947	33.142615	0.490692	C	17.305999	24.820216	2.521366
H	19.794174	20.609119	2.322089	C	16.395263	23.734887	2.346640
H	20.927445	21.994115	2.953181	C	20.558157	23.016344	2.951491
H	10.759002	22.230306	1.816063	C	21.470889	24.049533	3.192679
H	10.865415	28.165546	1.774970	C	20.994915	25.393530	3.178623
H	10.105098	23.977352	1.713935	C	19.607944	25.641431	2.958007
H	16.095158	31.008069	2.216005	C	16.823738	26.155597	2.514339
H	12.128232	29.591240	1.583346	C	15.434662	26.416441	2.319249
H	17.798948	30.636240	2.962078	C	14.525716	25.334557	2.174066
H	33.685424	25.700481	3.530799	C	15.005951	23.991267	2.196521
H	30.437242	28.415022	4.006462	C	13.118840	25.591128	2.010594
H	9.437249	14.882513	2.198336	C	12.193858	24.508171	1.923358
H	12.709532	12.164890	2.432579	C	12.690108	23.135790	1.963470
H	9.552199	20.811740	1.527732	C	14.088053	22.893458	2.075997
H	8.814263	16.619435	1.661044	C	17.737940	27.242540	2.718886
H	22.126587	18.593647	0.820817	C	17.271140	28.585945	2.680895

H	20.394012	18.935492	1.522400	C	15.872803	28.861175	2.367370
H	20.256409	7.086929	1.991996	C	14.958881	27.772677	2.241661
H	24.128741	8.512976	0.941691	C	11.825963	22.040506	1.883035
H	15.287998	10.225394	3.058273	C	14.566546	21.551114	2.073466
H	18.519582	7.471457	2.705660	C	12.634002	26.931240	1.937974
H	14.573423	11.879193	2.409236	C	11.254289	27.147689	1.808413
H	25.244676	9.951425	0.545366	C	10.341855	26.097075	1.725026
H	27.074246	9.579510	3.921395	C	10.826799	24.791315	1.784132
H	35.900601	22.425385	1.885266	C	15.401422	30.167637	2.173721
H	35.621352	20.668537	1.938104	C	14.062760	30.436628	1.884909
H	36.096990	21.538777	3.406305	C	13.168926	29.370095	1.818513
H	19.209498	34.291063	0.733811	C	13.579750	28.040347	2.003161
H	19.963777	34.712417	3.248582	C	19.118447	26.989734	2.965258
H	20.853900	34.946397	0.560905	C	20.017143	28.067575	3.216825
H	27.711714	33.477926	4.003663	C	19.518369	29.399305	3.252070
H	28.063005	32.684815	1.389874	C	18.169909	29.615573	2.963438
H	26.693106	33.820915	1.388706	C	31.729182	24.777010	3.497357
H	8.252575	25.546770	1.909187	C	32.609142	25.866105	3.587224
H	8.637748	26.494079	0.461769	C	32.155062	27.172003	3.776583
H	8.563142	27.294332	2.039533	C	30.781465	27.390537	3.865678
H	13.600017	32.435251	2.580672	C	29.850306	26.342394	3.790167
H	12.579952	31.872633	1.243201	C	32.802409	14.499991	3.335850
H	14.259707	32.372461	0.938996	C	29.093784	10.157746	3.536378
H	33.467758	28.635706	2.866643	C	17.864959	19.725981	2.057961
H	33.887344	28.048761	0.320922	C	16.459750	19.962374	2.067599
H	32.537028	29.205257	0.234898	C	12.260807	20.713607	1.892700
H	34.537556	14.125228	2.104846	C	13.657188	20.456617	1.984122
H	34.565037	13.287181	3.665757	C	16.028319	17.527575	1.968233
H	34.886163	15.036949	3.582947	C	15.539699	18.865416	1.999103
H	30.527222	8.731365	2.773019	C	14.141737	19.107430	1.988202
H	28.839449	8.173133	2.709347	C	13.226433	18.013192	1.978375
H	29.566392	8.217003	0.137302	C	13.714154	16.680302	2.017824
H	10.480494	11.397298	2.943423	C	15.119013	16.439691	2.004088
H	9.642780	11.936754	1.477963	C	12.793640	15.576160	2.081074
H	9.133154	12.556041	3.055903	C	11.811679	18.261508	1.912773
H	7.013010	18.967173	2.324934	C	11.386876	15.808376	2.049361
H	7.125180	18.219815	0.723729	C	10.514791	14.718548	2.181940
H	7.387417	19.969590	0.911171	C	10.981537	13.411262	2.325937
H	22.100659	5.595680	0.842098	C	12.358352	13.191132	2.323696
H	22.803431	5.816976	2.448947	C	13.281274	14.241195	2.198227
H	23.768025	6.192423	1.008039	C	11.320764	19.596693	1.818211
H	16.458313	6.923263	4.033451	C	9.942342	19.800910	1.646430
H	15.529085	7.031820	2.527599	C	9.037999	18.741760	1.584630
H	15.049738	8.004484	3.932177	C	9.526393	17.442473	1.723310

H	32.279509	12.431925	3.401241	C	10.890407	17.172917	1.898657
H	23.219940	19.981528	0.150801	C	23.843487	17.362501	0.486246
C	22.889203	23.790437	3.434348	C	22.498248	17.572676	0.803037
C	23.401009	22.493592	3.560171	C	21.605430	16.539139	1.102270
C	24.762419	22.217783	3.724556	C	22.079841	15.195660	1.052755
C	25.683215	23.304880	3.701116	C	23.450663	14.946749	0.753661
C	25.188390	24.639000	3.660960	C	24.340938	16.027423	0.486130
C	23.791842	24.888908	3.547153	C	20.208102	16.798561	1.439390
C	25.267921	20.857801	3.895635	C	19.315164	15.700372	1.604352
C	26.670789	20.620388	3.816340	C	19.806200	14.354331	1.557358
C	27.583419	21.719975	3.695678	C	21.184958	14.102094	1.298741
C	27.093737	23.058080	3.704988	C	19.707591	18.095541	1.604698
C	27.160827	19.284880	3.874269	C	18.358157	18.368821	1.850928
C	28.977693	21.477252	3.593057	C	17.436902	17.282258	1.878666
C	29.889963	22.570912	3.532773	C	17.927792	15.949275	1.803794
C	29.405537	23.903063	3.616538	C	18.918321	13.269002	1.780139
C	28.003749	24.147046	3.693228	C	19.408893	11.930752	1.786335
C	30.328172	25.008119	3.629931	C	20.781099	11.679015	1.525953
C	28.412360	26.584966	3.855426	C	21.667022	12.766598	1.264745
C	27.507123	25.490335	3.766530	C	21.275675	10.327633	1.521545
C	29.462622	20.128628	3.556559	C	22.640242	10.064964	1.208256
C	30.851749	19.874365	3.378402	C	23.046692	12.515495	0.966537
C	31.781317	20.990059	3.227556	C	17.522216	13.522602	1.981252
C	31.296007	22.324188	3.361468	C	16.623197	12.447071	2.215884
C	27.889309	27.872608	3.991329	C	17.142820	11.093840	2.362257
C	26.519943	28.146805	4.004780	C	18.519332	10.842573	2.094647
C	25.605278	27.069313	3.832321	C	23.937449	13.598119	0.710526
C	26.104592	25.738168	3.749215	C	20.406256	9.237564	1.829005
C	33.142972	20.789339	2.951145	C	20.913793	7.931851	1.784480
C	34.036012	21.851247	2.817154	C	22.247111	7.663145	1.467075
C	33.558318	23.150071	2.999057	C	23.090814	8.734434	1.188616
C	32.213092	23.414969	3.288022	C	16.325824	10.034726	2.784007
C	28.562912	19.034491	3.709971	C	16.809850	8.734063	2.910836
C	29.047531	17.694425	3.706377	C	18.145658	8.488945	2.591081
C	30.440063	17.456922	3.528995	C	19.012768	9.507626	2.172449
C	31.292160	18.550061	3.368991	C	17.017642	14.851856	1.940449
C	27.725458	14.175006	4.027362	C	15.619113	15.097763	2.042494
C	28.626313	15.257552	3.846865	C	14.721200	14.001922	2.179726
C	28.141586	16.597759	3.889733	C	15.255235	12.715868	2.286096
C	30.027083	15.004852	3.633149	C	23.534941	11.179328	0.915742
C	28.198838	12.820057	3.922123	C	24.873370	10.970728	0.584016
C	30.511237	13.666605	3.559455	C	25.766963	12.007190	0.310905
C	31.889668	13.449751	3.423131	C	25.305899	13.350146	0.400042
C	32.315061	15.805241	3.374487	C	35.486990	21.607636	2.491982

C	30.946880	16.088196	3.511416	C	20.109188	34.283099	0.100573
C	27.292935	11.728050	4.068849	C	27.192991	33.029547	0.813667
C	29.980259	11.229046	3.445115	C	8.873004	26.370819	1.529540
C	29.569934	12.555818	3.643033	C	13.600264	31.850208	1.648143
C	21.902163	26.484312	3.389183	C	33.129753	28.319915	3.865030
C	23.296807	26.235317	3.551080	C	34.273939	14.223251	3.169036
C	24.195955	27.319987	3.733450	C	29.565744	8.745953	3.303724
C	23.702627	28.655776	3.808225	C	10.011395	12.266226	2.461550
C	22.312787	28.902550	3.655718	C	7.566402	18.989116	1.373307
C	21.414704	27.817364	3.423856	C	22.758602	6.246184	1.437228
C	21.808384	30.246543	3.751161	C	15.914822	7.617588	3.376970
C	24.611676	29.746022	4.051222	C	27.761901	10.422716	3.857617
C	22.688195	31.328898	4.044805	C	24.283983	19.785198	0.037952
C	22.013695	32.629785	0.072200	C	24.742840	18.469439	0.169399
C	20.789550	32.875832	4.048646	C	26.268321	18.197027	4.091260
C	19.940164	31.812025	3.742337	C	26.761345	16.850702	4.111798
C	20.415359	30.503190	3.579003	C	25.726864	15.775279	0.216741
C	25.874035	29.515906	0.059915	C	26.209308	14.440801	0.172867

Supplementary References

1. Hayashi, S., Hayamizu, K. Chemical Shift Standards in High-Resolution Solid-State NMR (1) ^{13}C, ^{29}Si, and ^{1}H Nuclei. *Bull. Chem. Soc. Jpn.* **64**, 685-687 (1991).
2. Niu, L. T., Zhang, H., Yang, H. J., Fu, H. Metal-Free Iodination of Arylboronic Acids and the Synthesis of Biaryl Derivatives. *Synlett* **25**, 995-1000 (2014).
3. Ramesh, E., Guntreddi, T., Sahoo, A. K. $AlCl_3$-Catalyzed Intermolecular Annulation of Thiol Derivatives and Alkynes by 1,2-Sulfur Migration: Construction of 6-Substituted Benzo[*b*]thiophenes. *Eur. J. Org. Chem.* **2017**, 4405-4413 (2017).
4. Vo, T. H., Shekhirev, M., Kunkel, D. A., Orange, F., Guinel, M. J. F., Enders, A., Sinitskii, A. Bottom-Up Solution Synthesis of Narrow Nitrogen-Doped Graphene Nanoribbons. *Chem. Commun.* **50**, 4172-4174 (2014).
5. Hisaki, I., Nakagawa, S., Tohnai, N., Miyata, M. A C_3-Symmetric Macrocycle-Based, Hydrogen-Bonded, Multiporous Hexagonal Network as a Motif of Porous Molecular Crystals. *Angew. Chem., Int. Ed.* **54**, 3008-3012 (2015).
6. Klein, P., Lechner, V. D., Schimmel, T., Hintermann, L. Generation of Organozinc Reagents by Nickel Diazadiene Complex Catalyzed Zinc Insertion into Aryl Sulfonates. *Chem. Eur. J.* **26**, 176-180 (2020).
7. Yang, Q. L., Xing, Y. K., Wang, X. Y., Ma, H. X., Weng, X. J., Yang, X., Guo, H. M., Mei, T. S. Electrochemistry-Enabled Ir-Catalyzed Vinylic C–H Functionalization. *J. Am. Chem. Soc.* **141**, 18970-18976 (2019).
8. Alameddine, B., Anju, R. S., Al-Sagheer, F., Jenny, T. A. Tribenzopentaphene Derivatives with Lateral Aromatic Groups: The Effect of the Nature and Position of Substituents on Emission Properties. *New J. Chem.* **40**, 10363-10370 (2016).
