# Supplementary material for: Synthesis of a magnetic π-extended carbon nanosolenoid with Riemann surfaces
Source: Nat Commun. 2022 Mar 9;13:1239. doi: 10.1038/s41467-022-28870-z (PMC8907333; doi:10.1038/s41467-022-28870-z)
Supplement: Supplementary file 3 — Description of Additional Supplementary Files [file 41467_2022_28870_MOESM3_ESM.pdf]

Title: Supplementary Movie 1.
Description: Molecular dynamic simulation results at 300 K reveal the thermal stability of the bundles configuration.
