# Supplementary figures and images for: Synthesis of a magnetic π-extended carbon nanosolenoid with Riemann surfaces
Source: Nat Commun. 2022 Mar 9;13:1239. doi: 10.1038/s41467-022-28870-z (PMC8907333; doi:10.1038/s41467-022-28870-z)

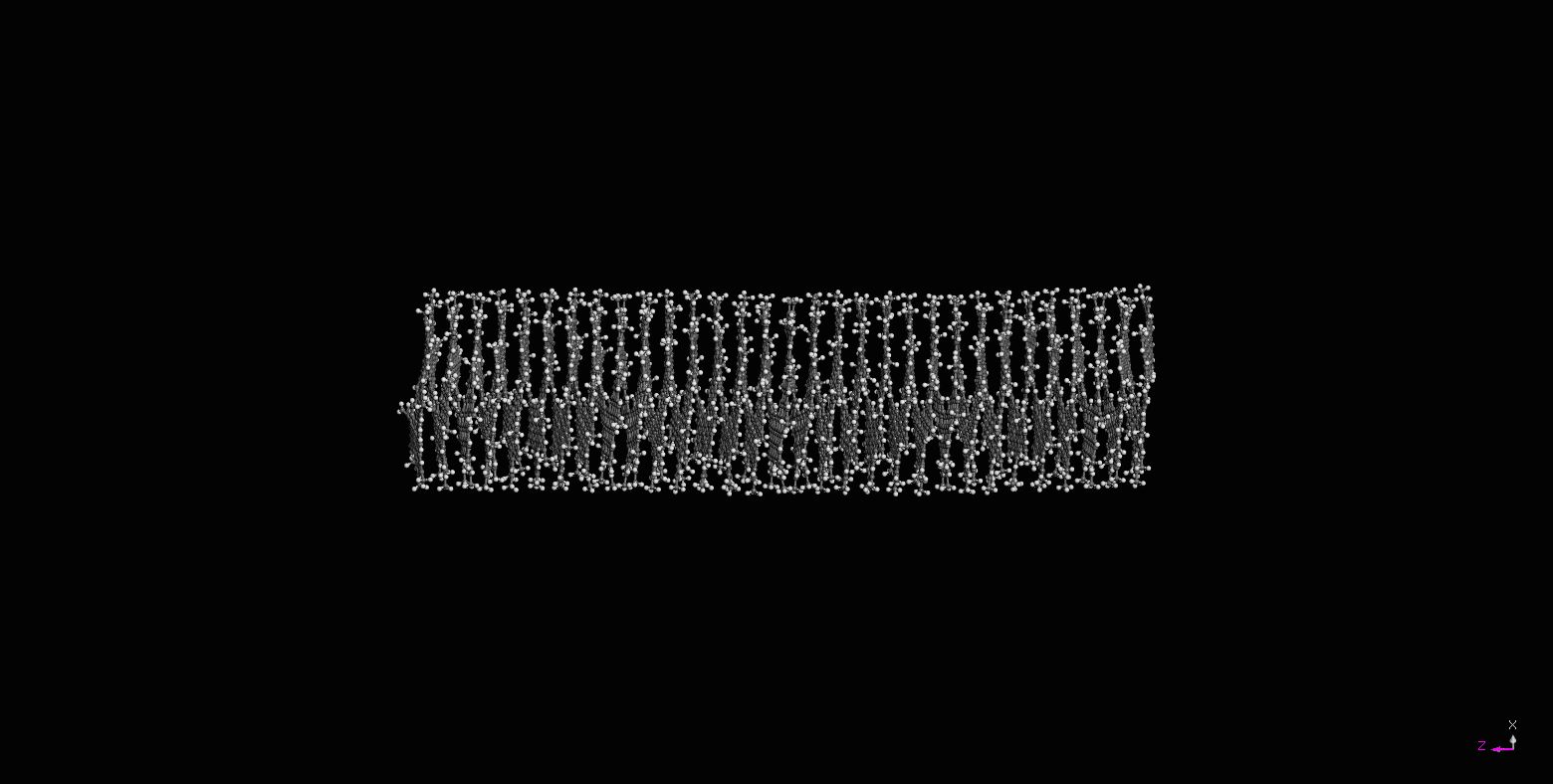

Supplement: Supplementary file 4 — Supplementary Movie 1 [file 41467_2022_28870_MOESM4_ESM.gif]
